# Glioblastoma PET/MRI: kinetic investigation of [^18^F]rhPSMA-7.3, [^18^F]FET and [^18^F]fluciclovine in an orthotopic mouse model of cancer

**DOI:** 10.1007/s00259-022-06040-z

**Published:** 2022-11-22

**Authors:** Marcel Lindemann, Ana Oteiza, Montserrat Martin-Armas, Yngve Guttormsen, Angel Moldes-Anaya, Rodrigo Berzaghi, Trond Velde Bogsrud, Tore Bach-Gansmo, Rune Sundset, Mathias Kranz

**Affiliations:** 1 PET Imaging Center Tromsø, University Hospital of North Norway (UNN), Tromsø, Norway; 2 Nuclear Medicine and Radiation Biology, UiT The Arctic University of Norway, Tromsø, Norway; 3 PET Center, Aarhus University Hospital, Aarhus, Denmark

**Keywords:** Glioblastoma, PET/MRI, Amino acid transport, PSMA, Neo-vascularization

## Abstract

**Purpose:**

Glioblastoma multiforme (GBM) is the most common glioma and standard therapies can only slightly prolong the survival. Neo-vascularization is a potential target to image tumor microenvironment, as it defines its brain invasion. We investigate [^18^F]rhPSMA-7.3 with PET/MRI for quantitative imaging of neo-vascularization in GBM bearing mice and human tumor tissue and compare it to [^18^F]FET and [^18^F]fluciclovine using PET pharmacokinetic modeling (PKM).

**Methods:**

[^18^F]rhPSMA-7.3, [^18^F]FET, and [^18^F]fluciclovine were i.v. injected with 10.5 ± 3.1 MBq, 8.0 ± 2.2 MBq, 11.5 ± 1.9 MBq (*n* = 28, GL261-luc2) and up to 90 min PET/MR imaged 21/28 days after surgery. Regions of interest were delineated on T2-weighted MRI for (i) tumor, (ii) brain, and (iii) the inferior vena cava. Time-activity curves were expressed as SUV mean, SUVR and PKM performed using 1-/2-tissue-compartment models (1TCM, 2TCM), Patlak and Logan analysis (LA). Immunofluorescent staining (IFS), western blotting, and autoradiography of tumor tissue were performed for result validation.

**Results:**

[^18^F]rhPSMA-7.3 showed a tumor uptake with a tumor-to-background-ratio (TBR) = 2.1–2.5, in 15–60 min. PKM (2TCM) confirmed higher K1 (0.34/0.08, *p* = 0.0012) and volume of distribution *V*_T_ (0.24/0.1, *p* = 0.0017) in the tumor region compared to the brain. Linearity in LA and similar k3 = 0.6 and k4 = 0.47 (2TCM, tumor, *p* = ns) indicated reversible binding. K1, an indicator for vascularization, increased (0.1/0.34, 21 to 28 days, *p* < 0.005). IFS confirmed co-expression of PSMA and tumor vascularization. [^18^F]fluciclovine showed higher TBR (2.5/1.8, *p* < 0.001, 60 min) and *V*_S_ (1.3/0.7, *p* < 0.05, tumor) compared to [^18^F]FET and LA indicated reversible binding. *V*_T_ increased (*p* < 0.001, tumor, 21 to 28 days) for [^18^F]FET (0.5–1.4) and [^18^F]fluciclovine (0.84–1.5).

**Conclusion:**

[^18^F]rhPSMA-7.3 showed to be a potential candidate to investigate the tumor microenvironment of GBM. Following PKM, this uptake was associated with tumor vascularization. In contrast to what is known from PSMA-PET in prostate cancer, reversible binding was found for [^18^F]rhPSMA-7.3 in GBM, contradicting cellular trapping. Finally, [^18^F]fluciclovine was superior to [^18^F]FET rendering it more suitable for PET imaging of GBM.

**Supplementary Information:**

The online version contains supplementary material available at 10.1007/s00259-022-06040-z.

## Introduction

The aggressive and lethal brain tumor, glioblastoma multiforme (GBM), is the most frequent form of glioma with an incidence of 3 per 100,000 in the USA [1]. The standard therapy, as described by the guidelines of the European Association of Neuro-Oncology [2] and the European Society for Medical Oncology [3], includes resection, chemotherapy, and irradiation [4]. Although the highest therapeutic goal is a complete resection during surgery, this is usually impossible due to its infiltrative growth. Resultantly, a relapse of the disease is inevitable, and the average patient survival is only increased to 15 months [4]. Some of the current MR sequences can detect metabolic changes within the tumor; however, in vivo metabolic characterization with high sensitivity can only be achieved by positron emission tomography (PET) [6, 7].

A promising radiotracer for GBM imaging with PET, [^18^F]fluciclovine, based on a synthetic leucine-derivate [8], shows an increased tumor-to-background ratio (TBR) compared to currently applied radiopharmaceuticals like [^18^F]FET [9]. Furthermore, it is superior to [^11^C]-methionine based on its low uptake in healthy brain tissue [10]. Different from naturally occurring amino acids, [^18^F]fluciclovine does not show radio metabolites nor incorporation into proteins and hence facilitates the data interpretation with regard to PKM [10–13].

The increased radiolabeled amino acid uptake is not directly correlated to higher protein synthesis of the growing tumor or to blood–brain barrier (BBB) breakdown but rather a measure of increased L-amino acid transporter (LAT) and alanine-serine-cysteine-transporter 2 (ASCT2) expression in tumor vasculature [14]. Hence, investigating the connection of tumor vasculature/progression with amino-acid-PET and neo-vascularization with specific PET radiopharmaceuticals open new insights into tumor diagnosis and new therapeutic options. Besides other emerging molecular targets, prostate specific membrane antigen (PSMA) expression by the neo-vascular endothelium was shown in a large variety of solid tumors including kidney, lung, breast, and pancreas cancer [15, 16]. In contrast, no expression in healthy endothelium and prostate cancer-associated neo-vasculature was found [17]. Although the mechanism is not fully understood yet, its contribution to cancer-related angiogenesis might be explained by its function degrading the extracellular matrix and by modulating the integrin signaling [15, 18]. Furthermore, recent studies provide evidence of a connection between radiolabeled PSMA ligand uptake in brain tumors and its PSMA expression on the microvascular endothelium of GBM neo-vasculature [19]. Hence, PSMA targeted therapies might improve GBM treatment by selectively destroying tumor vessels by high regional doses of drugs or internal radiotherapy [20].

A novel class of so called radiohybrid PSMA (rhPSMA) ligands is emerging [21]. The radiohybrid (rh) technology allows dual functionality using diagnostic or therapeutic radionuclides. Among them, [^18^F]rhPSMA-7.3 was originally developed for prostate cancer (PC) diagnostic imaging. Furthermore, it was recently investigated in a clinical study of PC with PET showing high uptake and favorable kinetics compared to current standard methods [22]. So far, [^18^F]rhPSMA-7.3 has not been considered for PET imaging approaches involving GBM.

In the present study, we investigated the utility of [^18^F]rhPSMA-7.3 for imaging of GBM related neo-vasculature in an orthotopic mouse model of GBM after 21 and 28 days of tumor growth in terms of kinetic uptake and binding characteristics. Furthermore, PET imaging of amino acid transporters was conducted to investigate its contribution to tumor vasculature/progression using PKM and the results compared to [^18^F]rhPSMA-7.3. Subsequently, possible advantages of the designated orphan drug [^18^F]fluciclovine over the commonly [7] used [^18^F]FET for GBM PET imaging were tested in the same setting confirming its suitability for diagnostic GBM PET.

## Materials and methods

### Animals

All animal experiments were approved by the Norwegian Food Safety Authority (Mattilsynet, #19743). C57BL/6JRj mice were purchased from Janvier (Le Genest-Saint-Isle, France) (female, 6–8 weeks) and housed in groups of 4 animals with free access to food, water and enrichment. The animals (*n* = 28) were allowed to adapt for 7 days before the start of experiments. The number of animals in this study was estimated a priori (G*Power 3.1), and 4 animals per group regarded sufficient to achieve a test power higher 90% (more details in supplemental information S2.3).

### Syngeneic orthotopic animal model of GBM

Following anesthesia (Isoflurane, 1.5% in oxygen), the mouse head was shaved, treated with iodine solution and ethanol, and fixed onto a stereotactic frame (Neurostar, Tübingen). The animals received a subcutaneous (s.c.) injection of meloxicam (5 mg/kg, 3 subsequent days), buprenorphine (0.1 mg/kg), and bupivacaine (1.5 mg/kg). A skin incision was made with a scalpel, exposing bregma and lambda. Using a drill, a bur hole was made −2.3 mm lateral and −0.5 mm anterior of bregma. A 10 µl Hamilton syringe filled with 3 µl of cell suspension (GL261-luc2) was inserted into the burr hole and 2 µl (5 × 10^4^ cells) injected over 10 min. The syringe was removed slowly after injection, the burr hole closed with bone wax and the skin sutured.

### Imaging of GBM and validation

The radiosynthesis of the three radiotracers (Fig. S1, Table S1) were performed in a semi- or fully automated process in a TRACERlab FX2 N (GE Healthcare, Chicago, USA) system, and procedures are described in detail in the Supplemental Information S2.1 as well as by Figs. S1–S11.

The animals were imaged weekly with 7 T magnetic resonance imaging (MRI) (PET/MRI, MR solutions, Guildford, UK) using a high-resolution mouse brain radiofrequency coil by applying T2-weighted fast spin echo (FSE) and diffusion-weighted imaging (DWI). The tumor volume was segmented (PMOD v.4.3, PMOD technologies) by placing a region of interest (ROI) around the hyper-intense lesion (Fig. S12) and the apparent diffusion coefficients (ADC) calculated from the slope of the linear regression of the signal intensity as a function of the *b* values in the tumor ROI (VivoQuant™ 2020, Invicro).

Simultaneous dynamic PET and MRI was performed using a dedicated 3-ring PET insert with 150 mm field of view for whole-body mouse imaging, depth of interaction crystal matrix, and 0.7 mm spatial resolution [23]. The mice were positioned prone in a mouse head bed (MINERVE, Esternay, France) and fixed with ear bars. The radiotracers were injected i.v. as a 150 µl bolus 21 and 28 days after tumor cell inoculation, 10.5 ± 3.1 MBq ([^18^F]rhPSMA-7.3, *n* = 5, 21 days; *n* = 6, 28 days), 11.5 ± 1.9 MBq ([^18^F]fluciclovine, *n* = 3, 21 days; *n* = 4, 28 days), and 8.0 ± 2.2 MBq ([^18^F]FET, *n* = 6, 21 days; *n* = 4, 28 days), and PET acquisition started for 90 min ([^18^F]rhPSMA-7.3: 60 min).

The list-mode data were reconstructed into 24 × 5 s, 8 × 60 s, (10) × 300 s or (16) × 300 s, 60 min, or 90 min time frames, respectively, using 3D ordered subset expectation maximization with 1 iteration, 32 subsets, and a VOXEL size of 0.42 mm, applying correction for random coincidences, decay, deadtime, and scatter correction.

For validation of the PET image-based results autoradiography, immunofluorescent staining and western blotting was performed as described in the Supplemental Information S2.4, S2.6, and S2.7. Written patient consent was obtained and approved by a local ethics committee (“Regional committee for medical and health research ethics” ID 295739).

### PET data analysis

The MRI-based ROI was used to define the metabolically active tumor volume (MTV) on the PET data by applying a threshold of 50% of the maximum ROI value (kBq/cm^3^) (MTV_50_) for [^18^F]FET/[^18^F]fluciclovine [24] and PET results expressed as mean SUV (SUVmean). Due to a general lower tumor uptake of [^18^F]rhPSMA-7.3, a threshold of 75% (MTV_75_) was used (Fig. S12). PKM was applied using the 1TCM, 2TCM, simplified reference tissue model, Logan and Patlak analysis (PMOD v.4.3, PMOD technologies), its suitability evaluated by Schwartz Criterion (SC), Akaike Information Criterion (AIK) and Model Selection Criterion (MSC). Furthermore, parametric maps (2TCM) were calculated for K1, k2, k3, k4, and *V*_T_.

The image derived input function (idIF) was segmented from the inferior vena cava as it provides a robust estimation of the idIF [25] with special regard to the spill-in contamination from neighboring tissue when using the heart [26]. Radiotracer specific model adaptation has been added as follows:

(i) [^18^F]rhPSMA-7.3, a plasma-to-blood ratio of 1.66, was chosen from the literature [27] and confirmed by in vivo experiments in mice. Although the radiotracer showed high metabolic stability during the investigation time, shown by radio-thin-layer chromatography (radio-TLC), radiometabolite correction was applied. Further details of in vivo metabolism data for [^18^F]rhPSMA-7.3 and radio-TLC are presented in the supplemental information Fig. S13 and S14.
(ii) [^18^F]fluciclovine is metabolically very stable, and no radiometabolite correction was applied [10–13]. Furthermore, according to the literature, no plasma-to-blood correction data was available [28].
(iii) [^18^F]FET, the plasma-to-blood ratio (1, 5, 15, 30, 40, and 55 min; 1.1, 1.1, 1.1, 1.1, 1.3, and 1.4) was chosen from the literature (confirmed for 30, 60, and 90 min by in vivo experiments in mice) and fitted in PMOD to correct the whole blood curve. Although high metabolic stability [29], the radiometabolite correction was applied based on in vivo stability studies in C57BL/6JRj mice and is in line with findings from Koopman et al. [30]. Further details of in vivo metabolism data for [^18^F]FET and radio-TLC are presented in the supplemental information Fig. S15 and S16.

To further investigate and decouple the uptake kinetics of the radiotracers, Patlak plots were used as a method for the investigation of binding compartments as linearity indicates irreversible uptake. Furthermore, early linearization in Logan plots is indicating reversible uptake in the regions under investigation. Finally, the simplified reference tissue model (SRTM) was evaluated as a possible tool for input-function-independent PKM (Table S4) by following the analysis method described by Koopman et al. [30]

The results were statistical evaluated with a *t* test (GraphPad Prism, v.9) and regarded significant for *p* ≤ 0.05.

## Results

### MRI monitoring

**Fig. 1:**
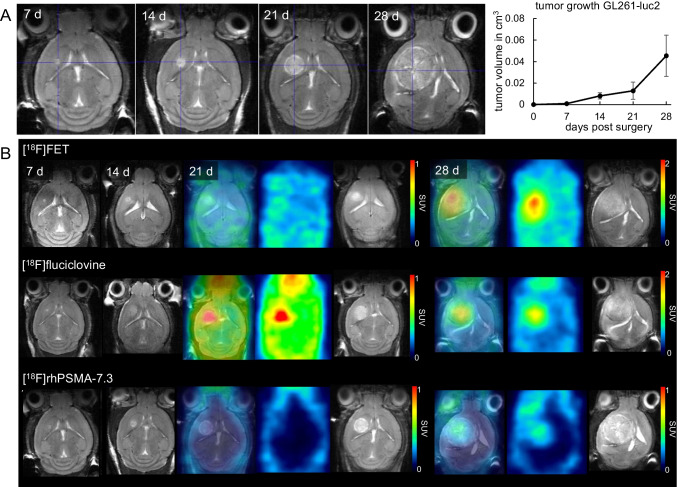
**A** T2-weighted FSE 7, 14, 21, and 28 days after GL261-luc2 cell inoculation visible in the left caudate putamen and its growth curve. **B** [^18^F]FET, [^18^F]fluciclovine, and [^18^F]rhPSMA-7.3 PET/MRI data of tumor bearing mice at different time points after tumor cell inoculation. Clear tumor uptake is visible at 28 days for all radiotracers

Following stereotactic surgery, the animals recovered quickly within 3 days. Weekly monitoring showed the tumor growth as hyper-intense signal on T2-weighted MRI in the left striatum (Fig. 1) up to 0.05 ± 0.02 cm^3^, 28 days after inoculation surgery.

Examples of ADC maps can be found in Fig. S17. The ADC value inside the tumor region increased significantly from 8.2*10^−4^ ± 2.7*10^−5^ to 9.15*10^−4^ ± 4.2*10^−5^ mm^2^/s, *p* = 0.03, day 21 to 28, indicating less restriction of diffusion inside the tumor area.

### Dynamic PET imaging

The automated radiosynthesis of the radiotracers showed good overall radiochemical yield, molar activities, and high radiochemical purities (see Table S1).

#### Semi-quantitative analysis

**Fig. 2:**
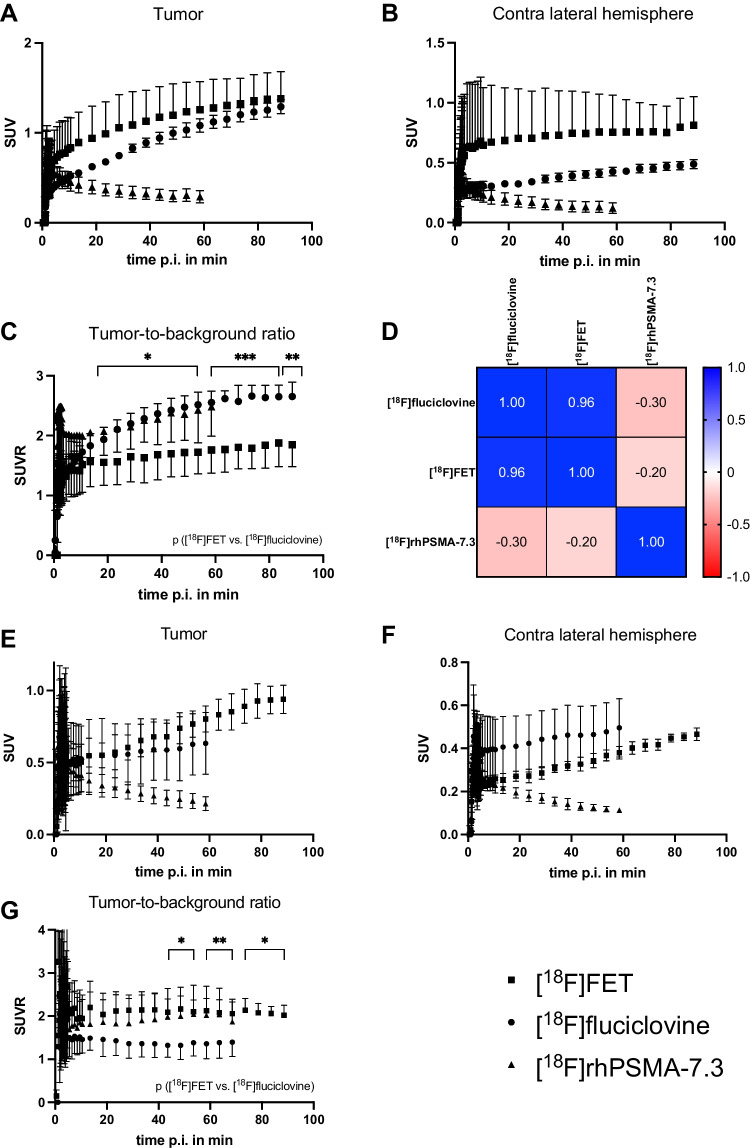
Dynamic uptake (SUVmean ± SD) following i.v. injection of [^18^F]FET (*n* = 4), [^18^F]fluciclovine (*n* = 4), and [^18^F]rhPSMA-7.3 (*n* = 6) after 28 days (**A–D**) or 21 days (**E–G**) of tumor growth in **A, E** the tumor region and **B, F** the contra lateral hemisphere. **C, G** TBR based on SUVmean using the contra lateral hemisphere as reference. **D** Heatmap of Pearson’s correlation (*r*) comparing tumor uptake of [^18^F]FET, [^18^F]fluciclovine, and [^18^F]rhPSMA-7.3. ^*^p < 0.05, ^**^p < 0.01, ^***^p < 0.001, *t* test

PET imaging with [^18^F]rhPSMA-7.3, [^18^F]fluciclovine, and [^18^F]FET showed a pronounced tumor uptake 28 days after surgery (Fig. 1). Unlike the two amino acid radiotracers, [^18^F]rhPSMA-7.3 showed an initial uptake (SUVmean 0.8, 2 min p.i.) followed by a washout phase until the end of the investigation (SUVmean 0.3, 60 min p.i.) (Fig. 2). Due to lower uptake in the other hemisphere (SUVmean 0.3, 4 min p.i.), [^18^F]rhPSMA-7.3 presented a similar TBR of 2.3 as [^18^F]fluciclovine, making the tumor visible by PET during the investigation time.

[^18^F]FET expressed the highest absolute tumor uptake (SUVmean 1.5, 90 min p.i.) (Fig. 2). Similarly, [^18^F]fluciclovine presented a constant radiotracer accumulation in the tumor, although reaching lower absolute SUVmean values (1.3, 90 min p.i.). However, the background uptake of [^18^F]fluciclovine in the other hemisphere was lower (0.5/0.8, 90 min p.i.) compared to [^18^F]FET (Fig. 2) resulting in significant higher TBR (2.7/1.9, *p* < 0.01) (Fig. 2). Only [^18^F]fluciclovine was able to show visible uptake 21 days (Fig. 2) after the inoculation surgery with a high TBR (2.0, 90 min p.i.). Resultantly, there was a high correlation (Pearson’s *r* = 0.96) between [^18^F]FET and [^18^F]fluciclovine uptake in the tumor region (Fig. 2).

#### Quantitative analysis

**Fig. 3:**
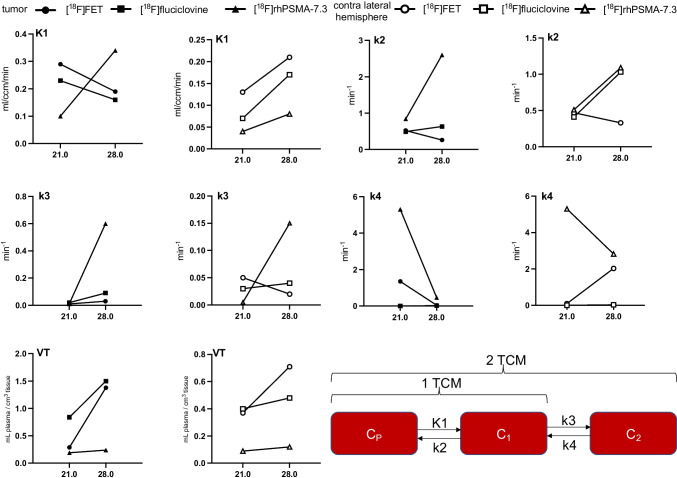
Kinetic parameters at 21 or 28 days post tumor cell inoculation using the 1TCM/2TCM according to the optimal evaluation parameters (Table S2/S3)

**Fig. 4:**
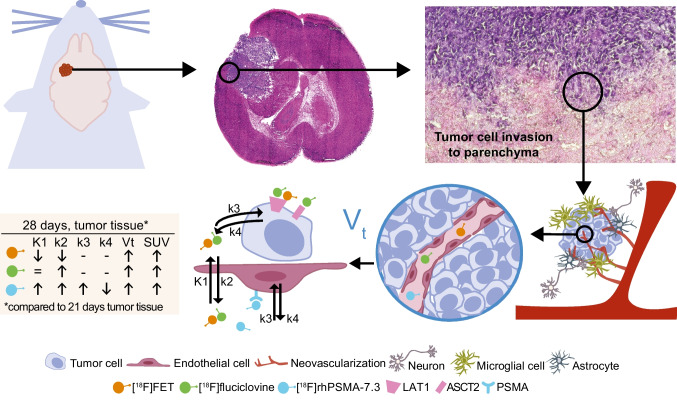
Overview over study design and suggested pathways. [^18^F]FET and [^18^F]fluciclovine are correlated with tumor growth and proliferation, and [^18^F]rhPSMA-7.3 is associated with endothelial cells of neo-vascularization. A summary of all PKM parameters is presented for its change over investigation time

Figure 3 and Tables S2 and S3 present all rate constants and macroparameters including evaluation parameters (mean values) and statistical *t* test results 21 and 28 days post tumor cell inoculation. The rate constants were expressed as ml/cm^3^/min (K1), 1/min (k2–k4) and ml/cm^3^ (*V*_T_, *V*s), and the results are summarized for the radiotracers in Fig. 4. Details of the graphical analysis and the SRTM can be found in the supplemental information (Fig. S18–S20 and Table S4).

#### [^18^F]rhPSMA-7.3

The kinetics are best described by the 2TCM. K1 (0.34/0.08, *p* = 0.0012) and *V*_T_ (0.24/0.1, *p* = 0.0017) were significantly higher for the tumor region as confirmed by parametric mapping (Fig. S21), too. K3, the transport of [^18^F]rhPSMA-7.3 to the receptor, was higher for tumor as for the healthy brain region (0.6/0.15, *p* = 0.31).

#### [^18^F]fluciclovine

The 2TCM described the kinetics of [^18^F]fluciclovine at 28 days after inoculation most accurately according to the SC and AIK. K1 (0.16/0.17, *p* = 0.4) and k2 (0.6/1.0, *p* = 0.6) were not significantly different between the tumor and brain region. However, k3 which describes the transport of [^18^F]fluciclovine into the tumor cell (via ASCT2/LAT1) was significantly higher (0.09/0.04, *p* << 0.001). *V*_T_ (1.5/0.5) and *V*_s_ (1.3/0.3) were significantly higher in the tumor region compared to the healthy brain (*p* << 0.001). For both, k3 and *V*_T_, parametric maps (Fig. S22) confirmed higher transport rates.

#### [^18^F]FET

The 2TCM described the kinetics of [^18^F]FET at 28 days best. *V*_T_ showed a significantly higher value for the tumor (1.38/0.71, *p* = 0.04) meaning the ratio of radiotracer in the tissue and the total parent fraction in plasma, which was confirmed by the parametric maps (Fig. S23).

Comparing [^18^F]FET to [^18^F]fluciclovine, [^18^F]fluciclovine expressed a higher TBR (Fig. 2) which is also described by the significant higher rate constants in the tumor *V*_s_ (1.26/0.7, *p* = 0.04), k3 (0.09/0.03, *p* = 0.0006), and k2 (0.6/0.3, *p* = 0.007).

### Validation of results

**Fig. 5:**
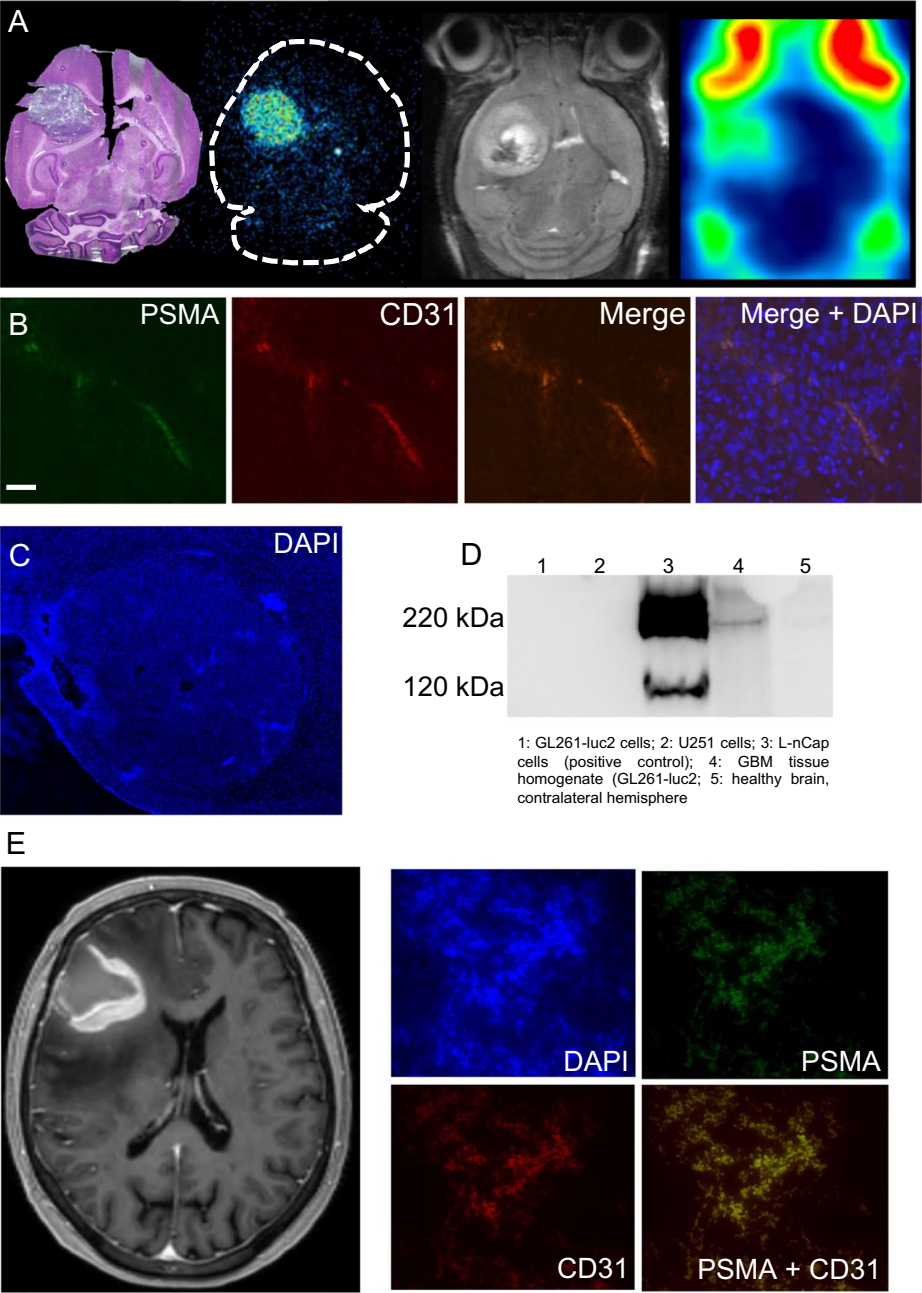
**A** H&E staining, autoradiography, T2-weighted MRI, and PET of GL261-luc2 tumor following injection of [^18^F]rhPSMA-7.3. **B** Immunofluorescent microscopy of GL261-luc2 brain tumor tissue (blue, DAPI/nuclei; red, CD31; green, PSMA), scale bar 50 μm, 40 × or **C** 2.5 × magnification showing the whole brain tumor. **D** Western blotting of cell lines and brain/tumor tissue, confirming PSMA expression in GBM tumor but not in GBM cell lines or healthy brain tissue. **E** MRI and corresponding immunofluorescent imaging of tumor tissue (blue, DAPI/nuclei; red, CD31; green, PSMA) of a patient with GBM

Ex vivo autoradiography of [^18^F]rhPSMA-7.3 confirmed tumor uptake and low uptake in the healthy brain (Fig. 5). Furthermore, immunofluorescent staining of mouse and human GBM confirmed co-expression of PSMA and the vascular endothelium marker CD31 in the tumor region but not in healthy brain. Western blotting confirmed PSMA protein expression in GL261-luc2 tumor tissue but not in GL261-luc2 cells and healthy brain.

## Discussion

We have performed PKM following application of a new PSMA specific radiotracer, [^18^F]rhPSMA-7.3, to investigate its suitability for imaging of the neo-vascularization in GBM and confirmed the findings by in vitro investigation using immunofluorescent imaging, western blot, and ex vivo autoradiography. Subsequently, two amino acid radiotracers, [^18^F]FET and [^18^F]fluciclovine, were tested in an orthotopic animal model of GBM 21 and 28 days after tumor cell inoculation.

Several reports have demonstrated that PSMA specific radiotracers are taken up by brain tumors [19] but not normal brain [31]. However, to date, no PKM has quantitatively evaluated the uptake kinetics of [^18^F]rhPSMA-7.3 in GBM. The uptake of [^18^F]rhPSMA-7.3 in the current study is reflected by both the 2TCM and Logan analysis, yielding identical and significant higher *V*_T_ for the tumor compared to the brain. Of note, *V*_T_ contains free, non-specific, and specific bound components [32] and hence cannot be fully assigned to neo-vasculature-related PSMA binding. K1, the transport from blood into the tissue, was significant higher in the tumor than in brain and hence includes the process of increased angiogenesis, tumor permeability, and BBB leakage due to its disruption [31, 33, 34].

Contrary to current PSMA radiotracers which show an increasing and irreversible uptake into PC [27], the TACs of the tumor in our model show an initial uptake, followed by a wash-out. K3 and k4, which are associated with radiotracer binding to the receptor and its cell internalization or dissociation [35], do not show significant differences. Hence, no trapping or irreversible binding of this radiotracer was present [36]. However, k3 presents higher values in tumor compared to healthy brain tissue which might describe the process of internalization [36]. As PSMA ligands are known to be internalized [37], k4 may describe here the reversibility of this process [38]. This is expected for [^18^F]rhPSMA-7.3 which binds reversibly to its respective target on the cell membrane, and following internalization, the receptor is recirculated to the cell surface [27]. Consistent with the results from the 2TCM, the Patlak plot confirms the absence of an irreversible compartment as neither linearity nor a positive slope was found. In contrast, the Logan plot showed linearity from early time points indicating reversible uptake [39]. PSMA expression presents different patterns in tumor cells compared to tumor-associated neo-vasculature [40] which might explain a different function of PSMA in the neo-vascular endothelium. Resultantly, this was confirmed by western blotting which shows no PSMA protein expression in GL261-luc2 cells but in resected tumor tissue. Furthermore, we confirmed in the same animal model and in human samples co-expression of PSMA with the vascular endothelia marker CD31. To the best of our knowledge, no data is available for PSMA ligand binding to endothelium and its internalization. Resultantly, we suggest a possible explanation that proteins necessary for PSMA internalization might not be expressed in tumor associated endothelial cells or that minimal internalization is taking place.

To date, only one publication investigates [^18^F]fluciclovine in brain tumors with PKM [10], but no comparison of rate constants to other amino acid PET radiotracers has been published. [^18^F]fluciclovine expresses high TBR, *V*_T_ and k3 in the tumor, in accordance with current clinical findings [10]. As both, LAT1 and ASCT2, are upregulated in brain tumors [41] and are transporters for [^18^F]fluciclovine [42], an increased influx was expected. This was shown by significant higher k3 (*p* = 0.0006) and V_S_ (*p* < 0.05) for [^18^F]fluciclovine than for [^18^F]FET which is a substrate for LAT2 only. Although both amino acid radiotracers express similar SUVmean, the TBR of [^18^F]fluciclovine is higher due to the lower uptake in normal brain tissue. While LAT transports large neutral amino acids into the brain, ASCT transfers non-essential neutral small amino acids into the endothelial cells [43, 44]. Contrary, Na^+^-depended amino acid transporters help to maintain the neutral amino acid concentration to a certain level [8, 43]. Resultantly, the low uptake in the brain might be expressed by the latter and describe an active efflux from this tissue [44]. This is further described by higher k2 than K1 values in both tumor and healthy brain, in accordance with others [45]. [^18^F]FET PET presented marked uptake of the radiotracer into the GBM at 28 days with high TBR and increasing SUVmean over time, as shown for other preclinical models [29]. The visibility in the PET image is also reflected by the significantly higher *V*_T_ of the tumor compared to the brain which is well in line with the findings from Bolcaen et al. [29]

From 21 to 28 days, *V*_T_, the perfusion independent concentration of the amino acid radiotracers, is significantly increasing indicating higher amino acid consumption and LAT/ASCT expression [30]. For [^18^F]FET, K1 is decreasing which is in line with findings for necrotic tumor areas [29]. An increase in necrosis and hence less restricted diffusion was confirmed by DWI MRI. As K1 is influenced by vascularization and angiogenesis [46], the drop from 0.55 to 0.19 ml/cm^3^/min might be explained by a loss of vascularization at this stage of tumor progression. McKelvey et al. [47] showed in the GL261 GBM model that the number of vessels increases during the first days before dropping until 14 days after inoculation [47]. Of note, they injected 20 times more cells; in consequence, our model reaches only half of the size (21 days: 12.9 mm^3^ vs. 20.4 mm^3^, our study or McKelvey et al. [47], respectively). We expected that this loss in vessels happen at later tumor stages (21 and 28 days). Resultantly, the loss of vascularization and reduced vessel lumen in the tumor microenvironment might explain our findings of lower K1 values for [^18^F]FET. Additionally, Zagzag et al. [33] reported that following the tumor cell inoculation, the initially hijacked brain vessels fail to undergo angiogenesis and subsequently involute which finally leads to a reduced number of vessels, necrosis, and lower K1. They found that following vascular apoptosis (7–21 days), neo-vascularization occurs in the advanced stage of the tumor 21–28 days post inoculation [33]. This is in line with our findings of increased K1 and *V*_T_ for [^18^F]rhPSMA-7.3 at 28 days where angiogenesis and thus increased neo-vascular PSMA expression take place. McKelvey et al. [47] reported an increasing number of immature blood vessels correlating with the observed higher *V*_T_ which might be related to binding of [^18^F]rhPSMA-7.3 to neo-vascularization. Concurrently, increased cell proliferation is maintained which is in line with increased *V*_T_ of [^18^F]FET/[^18^F]fluciclovine [47].

The SRTM was evaluated as a method for PKM independent from an idIF. Similar to what was found by Koopman et al. [30], the SRTM is not suitable for kinetic analysis of high grade brain tumors like GBM with [^18^F]FET as major model assumptions are violated: (i) Reference and target region should be represented by a 1TCM and (ii) the distribution volume is the same for target and reference region [48]. Further issues using the SRTM occur from the increased blood volume contribution in the tumor area and regional differences in transport across the BBB (disrupted in GBM) which has to be equivalent between tumor and reference region [30, 49].

Our study has some limitations: (i) The number of animals is rather small and statistical significance was not reached for all parameters which might improve with a higher number of subjects. (ii) The data acquisition and analysis is mostly image based. However, data of metabolism and plasma fraction was collected by in vivo experiments for [^18^F]rhPSMA-7.3 and [^18^F]FET. (iii) The idIF was derived from the inferior vena cava, and among the scientific literature, there is some controversy about the ideal model [50]. In our case, and different from 2-[^18^F]FDG studies, the left ventricle was not visible in the PET images; thus, vena cava provided the most reliable data, as shown by others too [25, 26].

## Conclusions

The investigation of [^18^F]rhPSMA-7.3, first time successfully applied in GBM PET, revealed visible tumor uptake. Following PKM, we suggest that this uptake is associated to neo-vascularization, while the number of mature vessels decreases, leading to necrosis. Imaging tumor angiogenesis using [^18^F]rhPSMA-7.3 with respect to tumor grading might be an interesting approach for future diagnostic PET in brain cancer. However, further research is needed to explain the mechanism behind its uptake, with special regard to the role of endothelial cells and the internalization of [^18^F]rhPSMA-7.3. Furthermore, the diagnostic efficacy of [^18^F]fluciclovine was investigated and compared to [^18^F]FET. Resultantly, [^18^F]fluciclovine showed more suitable imaging properties over [^18^F]FET based on higher TBR. Results from PKM confirmed higher rate constants and macro parameters when applying [^18^F]fluciclovine, rendering it more suitable for in vivo PET imaging of GBM.

## Supplementary Information

Below is the link to the electronic supplementary material.

Supplementary file1 (PDF 6850 kb)

## Data Availability

The datasets generated during and/or analyzed during the current study are available from the corresponding author on reasonable request.
