# Supplementary material for: Glioblastoma PET/MRI: kinetic investigation of [18F]rhPSMA-7.3, [18F]FET and [18F]fluciclovine in an orthotopic mouse model of cancer
Source: Eur J Nucl Med Mol Imaging. 2022 Nov 22;50(4):1183–94. doi: 10.1007/s00259-022-06040-z (PMC9931868; doi:10.1007/s00259-022-06040-z)
Supplement: Supplementary file 1 — Supplementary file1 (PDF 6850 kb) [file 259_2022_6040_MOESM1_ESM.pdf]

Glioblastoma PET/MRI - Kinetic Investigation of [^{18}F]rhPSMA-7.3, [^{18}F]FET and [^{18}F]fluciclovine in an Orthotopic Mouse Model of Cancer

Marcel Lindemann[1,2*,] Ana Oteiza[1,2], Montserrat Martin-Armas[1,2], Yngve Guttormsen[1,2], Angel Moldes-Anaya[1,2], Rodrigo Berzaghi[2], Trond Velde Bogsrud[1,3], Tore Bach-Gansmo[1], Rune Sundset[1,2*], Mathias Kranz[1,2*§]

[1] PET Imaging Center Tromsø, University Hospital North-Norway, Tromsø, Norway

[2] Nuclear Medicine and Radiation Biology, UiT The Arctic University of Norway, Tromsø, Norway

[3] PET-center, Aarhus University Hospital, Aarhus, Denmark

[*]Contributed equally

[§] Corresponding author: Mathias Kranz, Hansine Hansens vei 82, 9019 Tromsø, Norway. +47 980 80 625, mathias.kranz@unn.no, 0000-0002-5641-7396

S1. Supplemental Introduction 3
S2. Supplemental Materials and Methods 4
S2.1. Radiochemistry 4
S2.1.1. General 5
S2.1.2. General Preparation of the K[^{18}F]F/K_2CO_3/K_{222}-complex 7
S2.1.3. Automated radiosynthesis of [^{18}F]FET 7
S2.1.4. Semi-automated radiosynthesis of [^{18}F]fluciclovine 12
S2.1.5. Semi-automated radiosynthesis of [^{18}F]rhPSMA-7.3 15
S2.2 Cell culture 18
S2.3 PET/MR and ROI definition 19
S2.4 *Ex vivo* Autoradiography 20
S2.7 *In vivo* stability of [^{18}F]FET and [^{18}F]rhPSMA-7.3 20
S2.6 Western Blotting 21
S2.7 Fluorescence Immunohistochemistry 22
S3. Supplemental table and figures 23
S3.1 Radiochemistry 23

S3.2 In vivo stability of [^{18}F]FET and [^{18}F]rhPSMA-7.3 24

S3.3 Diffusion weighted imaging – ADC maps 26

S3.4 Graphical analysis (28 days). Logan and Patlak plots to differentiate reversibility 27

S3.5 PET kinetic modeling – parameter mapping 35

S4. Supplemental references 37

S1. Supplemental Introduction

After showing potential brain tumor related symptoms like fatigue, sleep-, pain-, seizures-, mood- and cognitive-function disturbance [1], the patient is referred for MR imaging of the brain with gadolinium contrast [2] for a first diagnosis and subsequent follow up. Several T1- and T2-weighted images (Fast Spin Echo (FSE), Fluid Attenuation Inversion Recovery) will provide a good definition of the tumor delineation and the surrounding oedema [3]. The tumor tissue will appear either hypo- or hyperintense, depending on the respective weighting and contrast agent. However, malignant areas can be found outside this definition making more sophisticated techniques necessary [3][4]. Hence, advanced functional imaging like diffusion-weighted MR (DWI), apparent diffusion coefficient (ADC) mapping or MR spectroscopy (MRS) have been demonstrated to provide important information for tumor phenotyping [5] and therapy monitoring [6]. While DWI correlates with cell density and tumor integrity, perfusion MRI provides hemodynamic information, and dynamic contrast enhanced T1-weighted MRI provides a measure of vascular parameters [7][8][9].

The most applied radiotracer in PET cancer diagnostics is 2-[^{18}F]FDG, a glucose analogue ideal for peripheral tumor detection [10]. However, it presents limited suitability in brain tumor imaging and characterization due to its high background level of glucose consumption in the cerebral cortex and therefore low tumor-to-normal tissue ratio [11][10]. In contrast,

labeled amino acids are of particular interest due to their low uptake in healthy brain tissue and increased accumulation in malignant transformation due to increased amino acid transport [12]. The European Association of Nuclear Medicine and the European Association for Neuro-Oncology are recommending amino acid PET for the evaluation of tumor recurrence and post-therapeutic monitoring as it is superior to MR investigation [13][14]. The fluorinated amino acid analogue [^{18}F]fluoroethyltyrosine ([^{18}F]FET) is widely used in clinical PET diagnostics of brain cancer and due to its following properties a suitable radiotracer. The tumor accumulation is due to increased transport across tumor blood vessels [15] and the radiotracer enters the cell via amino acid transporters [16]. Furthermore, the compound shows a relatively high *in vivo* stability (in human: 95% at 5 min and 87% at 120 min) [16] making continued blood sampling and radiometabolite correction unnecessary for pharmacokinetic modeling [17]. However, not all low grade glioma (WHO grade II) show [^{18}F]FET uptake to the same extent and about one third are [^{18}F]FET negative [18] in contrast to high grade glioma (WHO grade III and IV) where 96% show uptake [19] [20].

S2. Supplemental Materials and Methods

S2.1. Radiochemistry

The radiosynthesis of the three radiotracers were performed in a semi- or fully automated TRACERlab FX2 N (GE Healthcare, USA) system.

The [^{18}F]FET radiosynthesis follows mainly the described procedure of Bourdier et al. [21], by a first nucleophilic radiofluorination step and an acidic deprotection to form the final product. The process was adjusted to the TRACERlab FX2 N system. For the preparative HPLC separation of the final product a mobile phase system containing of 7% ethanol (EtOH) and 93% 5 mM sodium phosphate buffer (pH = 6) was used. The product fraction was neutralized with sodium hydroxide to result in a final pH of ~7.5 which was used for animal injection. (See figures S 1 and S 2)

[^{18}F]fluciclovine is nowadays mainly produced in GE FASTlab systems [22][23]. We transferred the GE FASTlab protocol (internal protocol provided from Blue Earth Diagnostics Ltd.) to our TRACERlab FX2 N system, by reducing reaction- and final volumes, to use the final radiotracer solution for animal applications. The production of the [^{18}F]fluciclovine is performed by a first nucleophilic radiolabeling step, followed by a deprotection and cartridge purification to yield the final radiotracer. Quality control was performed via radio-TLC, which did not allow a determination of the molar activity. (Figures S 1 and S 6)

The synthesis of [^{18}F]rhPSMA-7.3 was adapted from literature [24] and internal protocols provided by Blue Earth Diagnostics Ltd. Besides the adaptation to the GE TRACERlab FX2 N system, using a semi-automated one-pot-one-step radiosynthesis approach, a volume reduction of the final product and pH adjustment (pH ~7.9) were needed, to result in [^{18}F]rhPSMA-7.3 suitable for animal injection. (Figures S 1 and S 9)

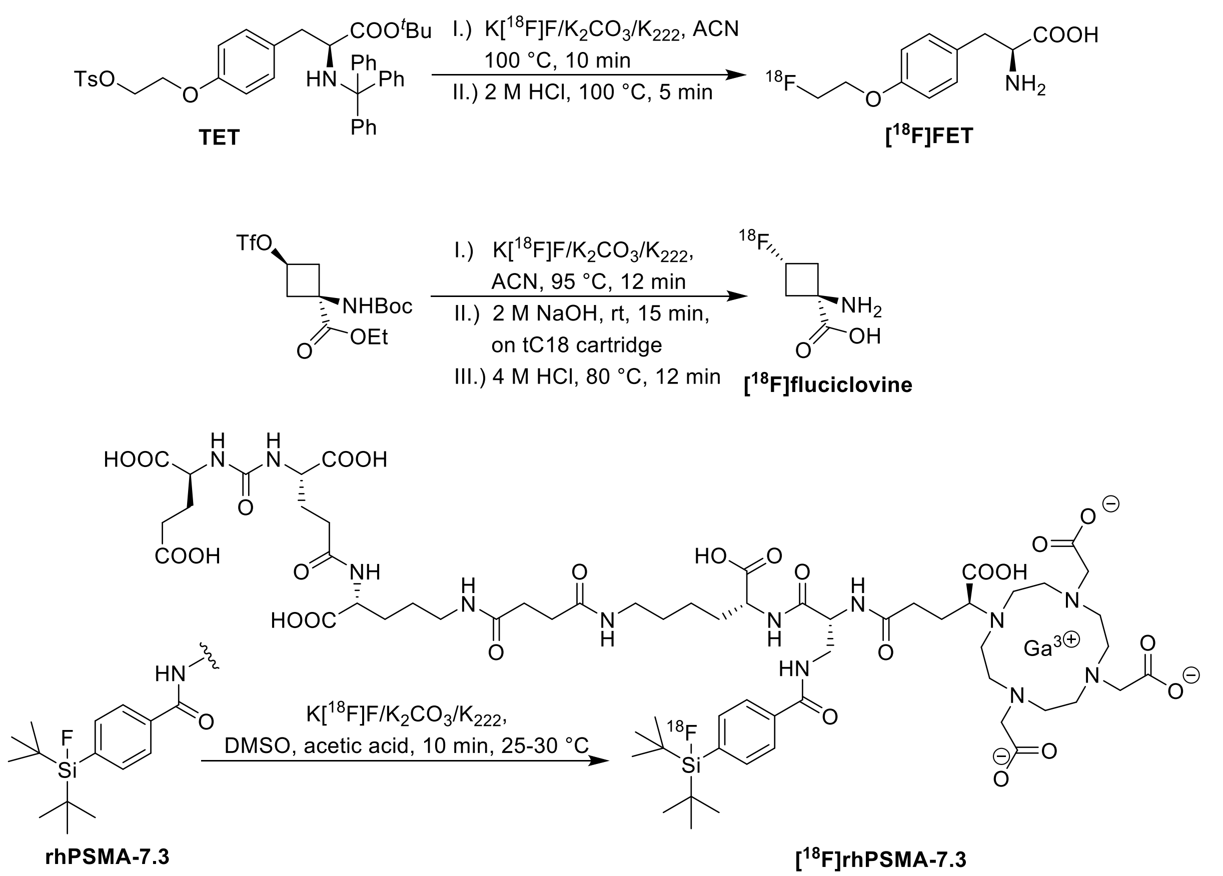

FIGURE S1: Radiosynthesis of [^{18}F]FET, [^{18}F]fluciclovine, and [^{18}F]rhPSMA-7.3.

S2.1.1. General

No-carrier-added [^{18}F]fluoride was produced via the [^{18}O(p,n)^{18}F] nuclear reaction by irradiation of a liquid target filled with H_2[^{18}O]O (Rotem Medical, Mishor Yamin, Israel) on a PETtrace 800-series cyclotron (15.5/8.4 MeV, GE Healthcare, Uppsala, Sweden).

The radiosynthesis of the different radiotracers was performed on the remote-controlled system TRACERlab FX2 N synthesizer (GE Healthcare, Waukesha, WI, USA) equipped with a Laboport vacuum pump (LABOPORT, KNF, Germany), a UV detector (Knauer DETECTION 10D, Berlin Germany) and was controlled with the TRACERlab FX2 Software (Version 2.3.0, GE Healthcare, Waukesha, WI, USA).

The radiosynthesis of the different radiotracers was performed with the following solid-phase extraction cartridges (preconditioning procedure): QMA – Sep-Pak Accell Plus QMA Plus Light cartridge, 130 mg sorbent, 37-55 µm (WAT023525, Waters Ltd., preconditioned with 10 ml EtOH, 10 ml 0.5 M aq. K_2CO_3 solution, 10 ml water, and 10 ml air); HLB – Oasis HLB

Plus LP extraction cartridge, 225 mg sorbent, 60 µm (186000132, Waters Ltd., preconditioning described below); tC18 – Sep-Pak tC18 Plus Short cartridge, 400 mg sorbent, 37-55 µm (WAT036810, Waters Ltd., preconditioned with 1 ml acetonitrile (ACN), 10 ml water, and 10 ml air); Alumina – Sep-Pak Alumina N Plus Light cartridge, 280 mg sorbent, 50-300 µm (WAT023561, Waters Ltd., preconditioned with 10 ml water and 10 ml air); and the C18 – Sep-Pak C18 Plus Light cartridge, 130 mg sorbent, 55-105 µm (WAT023501, Waters Ltd., preconditioned with 10 ml EtOH, 10 ml water and 10 ml air).

Analytical chromatographic separations were performed on an HPLC System from Agilent Technologies Infinity II 1260 series, with a sample injector (vial sampler, G7129A), pump (Quat. pump VL, G7111A), column oven (1260 MCT, G7116A; at 20 °C), UV detector (DAD WR, G7115A), and radio detector (NaI Detector, 1'' NaI/PMT, PMT3-0518007; Scan-RAM SR-1B, SR1B/0318/495, Lab Logic Systems Limited). Data were analysed with the HPLC software Open LAB CDS Chem Station Ed. Rev. C.01.08[210] (Agilent Technologies). Analytical separations were performed with solvent mixtures of MeOH, ACN, water, and 100 mM aq. ammonium formate (not pH adjusted), on an XSelect CSH C18 column (150 x 4.6 mm, 3.5 µm, Waters, Ireland) or on a Luna C18(2) column (100 x 4.6 mm, 3 µm, Phenomenex Ltd., Germany) at room temperature.

For radio-thin-layer chromatography (radio-TLC) silica gel G60 plates with fluorescence indicator F254 (Xtra SIL G/UV_{254} aluminium sheets, 50 x 100 mm, 200 µm, 60 Å, Macherey-Nagel GmbH & Co. KG, Germany) used, for non-radioactive detection dipping of the TLC plate for one second into a 0.9% ninhydrin staining (0.1 g ninhydrin in 10 ml EtOH and 100 ml water), and heating of the TLC plate afterwards. For detection of radioactive compounds, the radio-TLC scanner Scan-RAM SR-1B (SR1B/0318/495, with a PS Detector, PS Plastic/PMT, PMT2-0418007, LabLogic Systems Ltd., Broomhill, UK) was used. Compound R_f-values were calculated as ratio as the distance of compound to the total solvent front, radio-TLC was evaluated with the Laura-Radio HPLC, TLC, and GC chromatography software (Version 5.0.9.74SP8, LabLogic Systems Ltd., Broomhill, UK).

All chemicals were commercially purchased, unless noted. Solvents and reagents were used in HPLC grade or higher purities. The different precursor compounds and non-radioactive standards *O*-(2-tosyloxyethyl)-*N*-trityl-*L*-tyrosine tert-butyl ester (TET; CAS: 478037-15-9; Prod.-No. 3050.0025), syn-1-(*N*-(*tert*-butoxycarbonyl)amino)-3-[((trifluoromethyl)sulfonyl)oxy]cyclobutane-1-carboxylic acid ethyl ester (CAS: 939026-08-1; Prod.-No. BE-101-V3a), and FET · HCl ((2*S*)-*O*-(2'-fluoroethyl)-tyrosine hydrochloride;

Prod.-Nr. 3061.0005) were purchased from ABX advanced biochemical compounds GmbH (Radeberg, Germany). The non-radioactive rhPSMA-7.3 · TFA (CAS: 2305081-62-1), the (1r, 3r)-1-amino-3-fluorocyclobutanecarboxylic acid hydrochloride FACBC (fluciclovine) · HCl (CAS: 1033700-93-3), and the fluciclovine precursor (syn-1-(*N*-(*tert*-butoxycarbonyl)amino)-3-[((trifluoromethyl)sulfonyl)oxy]cyclobutane-1-carboxylic acid ethyl ester, CAS: 939026-08-1) were a gift from Blue Earth Diagnostics, UK.

S2.1.2. General Preparation of the K[^{18}F]F/K_2CO_3/K_{222}-complex

The cyclotron produced [^{18}F]fluoride in H_2[^{18}O]O was trapped on a QMA cartridge. The trapped [^{18}F]fluoride was eluted into reactor 1, with 2.0 ml of a K_2CO_3/K_{222} solution (1.7 mg K_2CO_3 and 9.5 mg K_{222} in 0.08 ml water and 1.92 ml ACN), the azeotropic drying process took place for 2 min at 65 °C under helium stream and vacuum, and afterward, 7.5 min at 95 °C in vacuum, followed by cooling to 25-30 °C.

S2.1.3. Automated radiosynthesis of [^{18}F]FET

To the before prepared K[^{18}F]F/K_2CO_3/K_{222}-complex the precursor TET (3.6-4.3 mg) dissolved in 2.0 ml ACN added to reactor 1 and the radiolabeling was performed at 100 °C for 10 min. After cooling to 50 °C, 1.0 ml 2 M aq. HCl was added, and heated to 100 °C for 3 min. Afterwards, a part of the ACN was evaporated at 100 °C under a stream of helium for 2 min. The reactor 1 was cooled to 35 °C, the reaction mixture was neutralized with a mixture of 0.5 ml 4 M aq. NaOH, 1.0 ml water, and 1.0 ml 5 mM aq. sodium phosphate buffer (pH = 6.0; 0.79 g $NaH_2PO_4 \cdot 2\ H_2O$ in 1 l water, pH adjusted with 1 M NaOH to a final pH = 6.0-6.1). The crude reaction mixture was filtered through a 0.45 µm PTFE syringe filter (d = 25 mm, VWR, 514-0071; preconditioned with 10 ml EtOH, 10 ml water, and 10 ml air) and injected onto the preparative HPLC system (Phenomenex Luna C18(2), 5 µm, 100 Å, 250 x 10 mm, Phenomenex Ltd., Germany; at a UV absorbance of 226 nm), with an eluent mixture of 7% EtOH/93% 5 mM aq. sodium phosphate buffer (pH = 6.0) with a flow rate of 4.0 ml/min. The product fraction was collected between 15.0-16.5 min. For injection into animals, the final [^{18}F]FET solution was pH adjusted with 1 M aq. NaOH to a final pH of ~ 7.5 .

Analytical HPLC was used for determination of the radiochemical purity, using the XSELECT analytical column with a linear gradient system of MeOH/water (0-1 min 5% MeOH, 1-17.5 min 5-95% MeOH, 17.5-20 min 95% MeOH, 20-21 min 95-5% MeOH, 21-23 min 5% MeOH; at 226 nm UV absorbance, flow rate 0.7 ml/min).

Molar activities (decay corrected to the EOS) were determined with a calibration curve and the non-radioactive standard FET · HCl using an isocratic HPLC method (10% MeOH/90% water, flowrate 1.0 ml/min, at the analytic HPLC system), using chromatograms obtained at 226 nm as an appropriate maximum of UV absorbance.

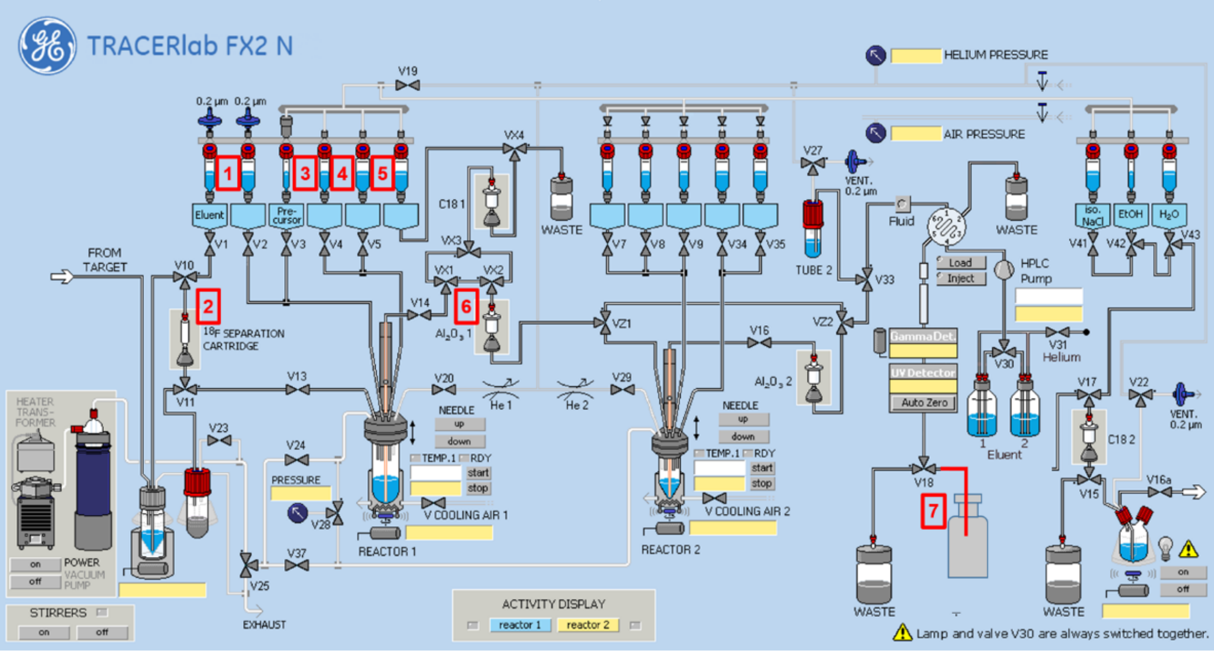

FIGURE S2: Synthesis module TRACERlab FX2 N (GE Healthcare) including the hardware modifications for the radiosynthesis of [^{18}F]FET (entry 1: 2.0 ml K_2CO_3/K_{222} ACN/water solution; entry 2: QMA cartridge; entry 3: TET in 2.0 ml ACN; entry 4: 1.0 ml 2 M aq. HCl; entry 5: 0.5 ml 4 M aq. NaOH, 1.0 ml water, and 1.0 ml 5 mM aq. phosphate buffer (adjusted pH = 6.0); entry 6: PTFE filter; entry 7: connection to the final vial or a falcon tube).

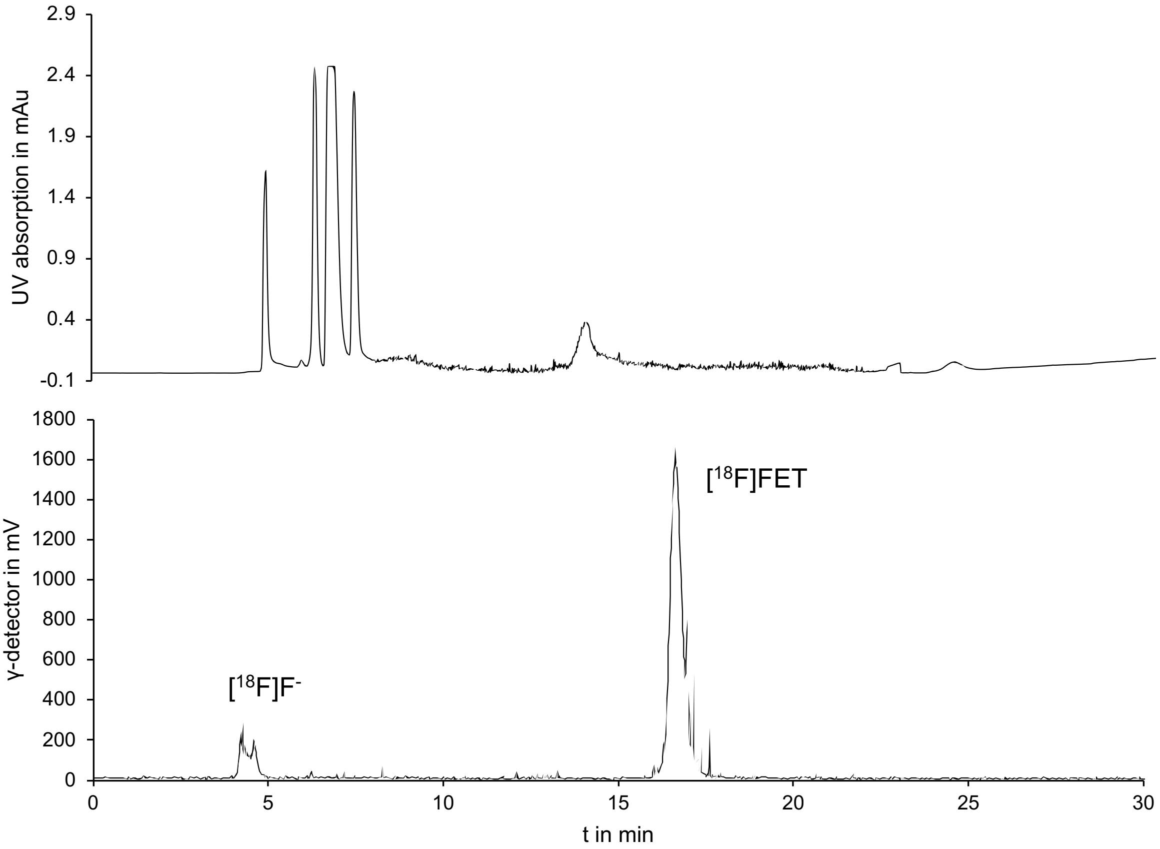

FIGURE S3: Preparative UV- and radio-HPLC chromatograms of [^{18}F]FET (conditions: Phenomenex Luna C18(2), 5 µm, 100 Å, 250 x 10 mm, Phenomenex Ltd., Germany; isocratic method 7% EtOH/93% 5 mM aq. sodium phosphate buffer (pH = 6) with a flowrate of 4 ml/min; at 226 nm)

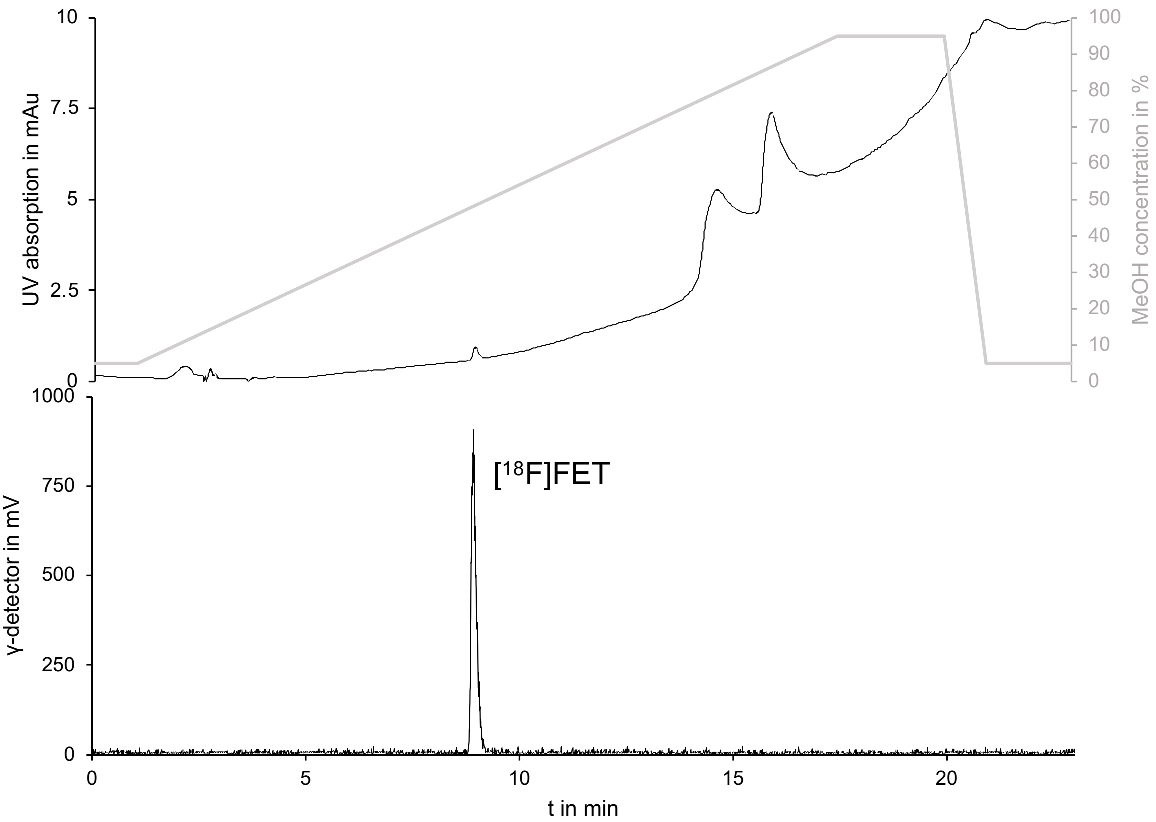

FIGURE S4: Analytical UV- and radio-HPLC chromatograms of the final product [^{18}F]FET (conditions: linear-gradient of MeOH/water (grey) with a flow rate of 0.7 ml/min; at 226 nm)

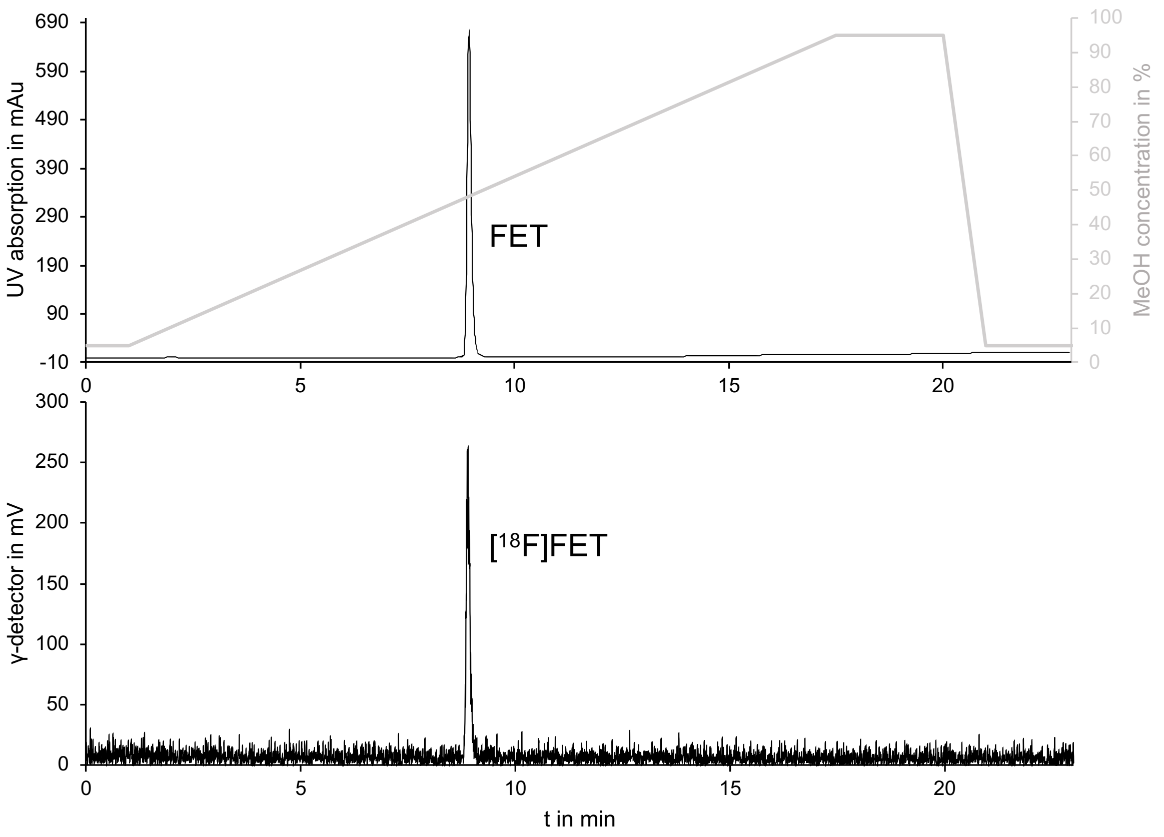

FIGURE S5: Analytical UV- and radio-HPLC chromatograms of the final product [^{18}F]FET spiked with the non-radioactive reference FET · HCl (conditions: linear-gradient of MeOH/water (grey) with a flow rate of 0.7 ml/min; at 226 nm)

S2.1.4. Semi-automated radiosynthesis of [^{18}F]fluciclovine

Without cooling of the prepared K[^{18}F]F/K_2CO_3/K_{222}-complex, the precursor (12.0-15.4 mg) dissolved in 1.0 ml ACN was added to reactor 1 and the radiolabeling was performed at 95 °C for 12 min. After cooling to 40 °C, the labeling mixture was diluted with 10 ml water and passed through the tC18 cartridge. After washing the cartridge twice with 10 ml water each wash, the cartridge was removed from the automated system, 0.5 ml 2 M aq. NaOH was pushed into the cartridge, and the hydrolysis took place for 15 min at room temperature. Afterwards, the NaOH was passed completely through the cartridge and the cartridge was flashed with 10 ml air. The NaOH solution was discarded and the tC18 cartridge placed back in the automated system, the radiolabeled intermediate was eluted from the cartridge with 1.5 ml EtOH into reactor 2. The EtOH was removed at 80 °C (6.5 min with a stream of helium and vacuum; 1.0 min only vacuum), then 0.5 ml 4 M aq. HCl was added, and heating continued at 80 °C for 12 min. After cooling to 30 °C and dilution with a mixture of 0.4 ml 4 M aq. NaOH and 0.6 ml water, the mixture was passed through an Alumina N, C18, and an HLB (preconditioned with 10 ml water and 10 ml air) cartridge into the final dose vial. The final formulation was pH adjusted with 1 M aq. HCl and 4.2% aq. sodium bicarbonate solution to a final pH of ~6.2.

Product identity and radiochemical purity were determined by radio-TLC with fluciclovine · HCl as non-radioactive standard. The final product shows a R_f value of ~0.67 (SiO_2; ACN/MeOH/water/conc. acetic acid 20 : 5 : 5 : 1 v/v/v/v).

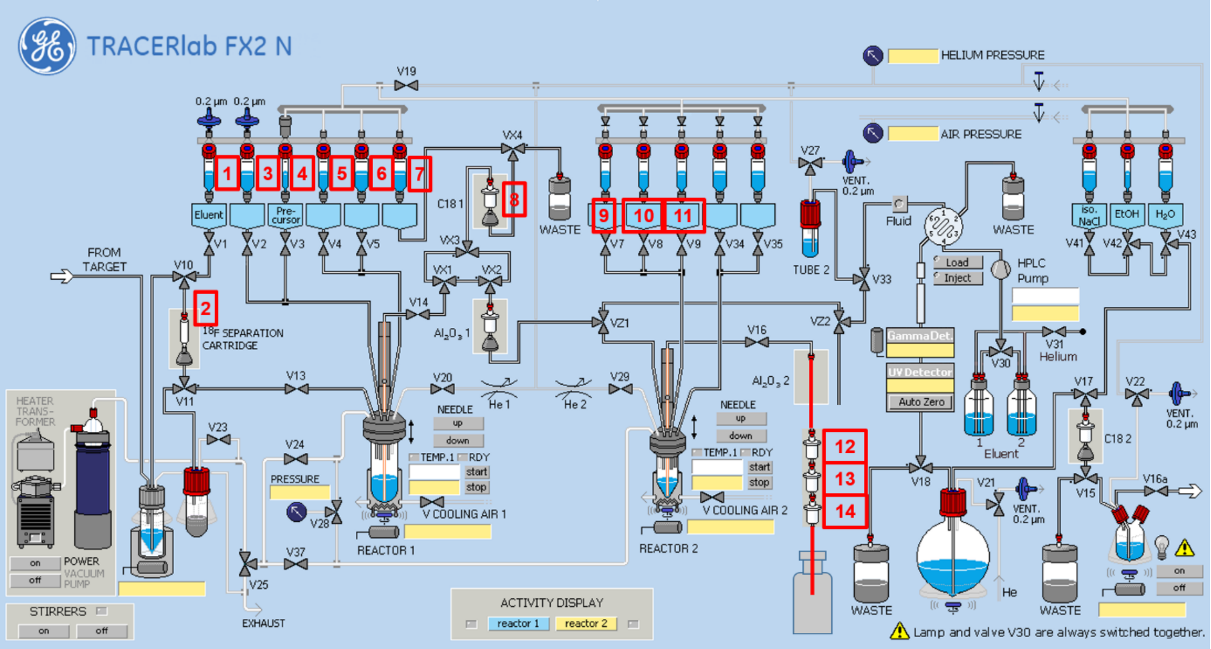

FIGURE S6: Synthesis module FX2 N (GE Healthcare) with the hardware modifications for the radiosynthesis of $[^{18}F]$fluciclovine (entry 1: 2.0 ml K_2CO_3/K_{222} ACN/water solution; entry 2: QMA cartridge; entry 3: 10 ml water; entry 4: precursor in 1.0 ml ACN; entry 5: 10 ml water; entry 6: 10 ml water; entry 7: 1.5 ml EtOH; entry 8: tC18 cartridge; entry 9: 0.5 ml 4 M aq. HCl; entry 10: 0.4 ml 4 M aq. NaOH and 0.6 ml water; entry 11: 2.0 ml water; entry 12: Alumina cartridge; entry 13: C18 cartridge; entry 14: HLB cartridge connected to the final dose vial).

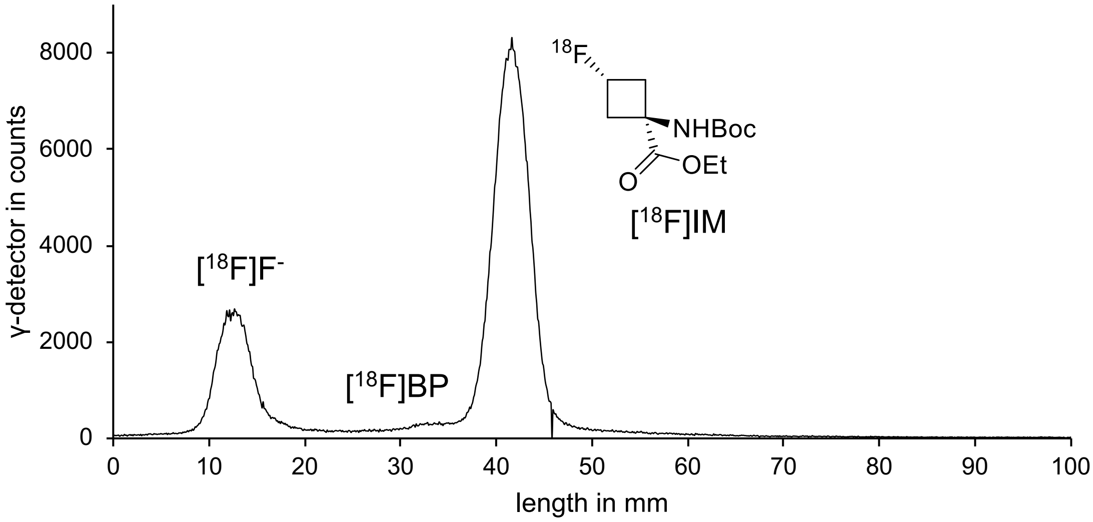

FIGURE S7: Radio-TLC chromatogram of the labeling solution (BP – byproduct; IM – protected [^{18}F]fluciclovine; conditions: SiO_2; ethyl acetate/*n*-hexane 1 : 5 v/v)

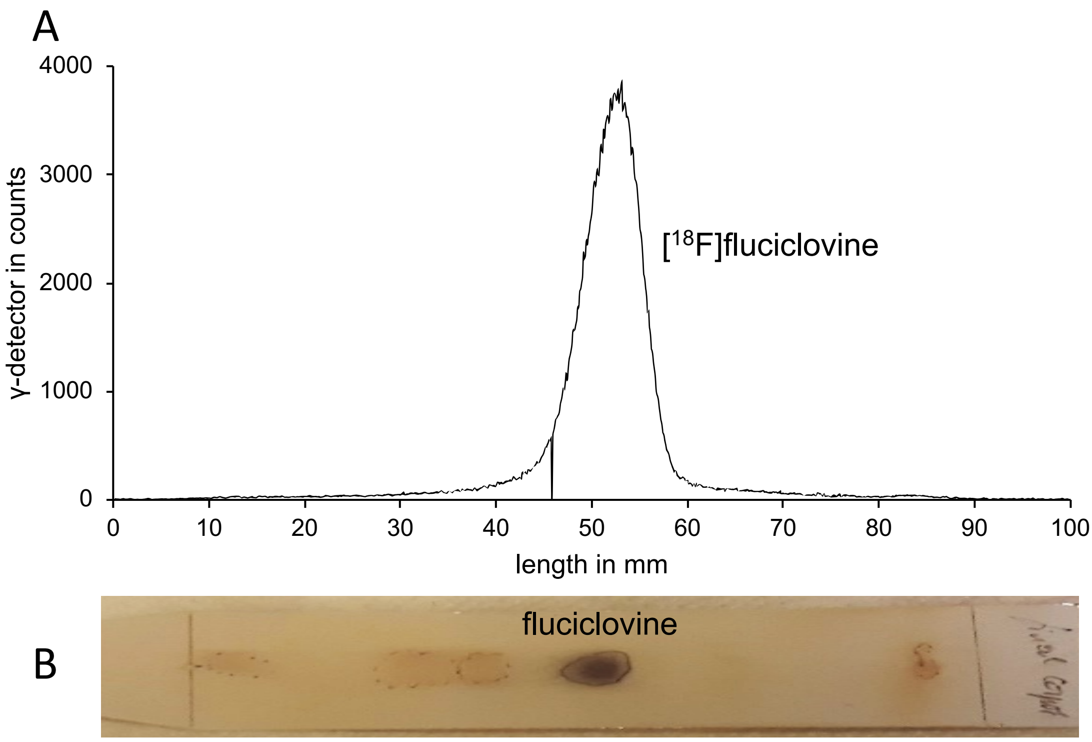

FIGURE S8: Radio-TLC chromatogram A) and radio-TLC plate co-spotted with fluciclovine · HCl; B) after staining with 0.9% ninhydrin solution of the final product [^{18}F]fluciclovine (conditions: SiO_2; ACN/MeOH/water/conc. acetic acid 20 : 5 : 5 : 1 v/v/v/v).

S2.1.5. Semi-automated radiosynthesis of [^{18}F]rhPSMA-7.3

To the before prepared K[^{18}F]F/K_2CO_3/K_{222}-complex (cooled to 25-30 °C), the precursor rhPSMA-7.3 · TFA (0.15-0.4 mg) dissolved in a mixture of 1.0 ml DMSO and 8.5 µl conc. acetic acid, added. The radiolabeling was performed at 25-30 °C for 10 min. After dilution with 10 ml 0.1 M aq. sodium acetate buffer (NaOAc; adjusted with conc. acetic acid to a final pH = 5) and transfer over the inlet connection of position "Al_2O_3 1" to the "C18 2" position (see Figure S 9 entry 7) equipped with an HLB cartridge (preconditioned with 10 ml ethanol, 10 ml water and 10 ml air). The second wash of the reactor was performed with 10 ml 0.1 M aq. sodium acetate NaOAc buffer, and the final washing step was performed from vial 6 with 5 ml 0.1 M aq. NaOAc buffer direct to the HLB cartridge ("C18 2"). After drying of the cartridge for 0.5 min, the inlet from "Al_2O_3 1" was removed and the original "C18 2" inlet was connected. The final product was eluted from the cartridge with two portions of 1.0 ml 50% aq. EtOH. The ethanolic solution was transferred to a V-vial and the volume was reduced at 40 °C with a slight vacuum and a stream of nitrogen for around 12 min to a final volume of ca. 1.0 ml. For injection into animals, the pH adjustment was performed with the addition of ca. 100 µl 4.2% aq. sodium bicarbonate solution to a final pH of ~7.2.

Analytical HPLC was used for the determination of the radiochemical purity, using a linear gradient system of ACN/100 mM aq. ammonium formate buffer (not pH adjusted; 0-2.5 min 5% ACN, 2.5-15 min 5-95% ACN, 15-17.5 min 95% ACN, 17.5-18 min 95-5% ACN, 18-20 min 5% ACN; at 240 nm UV absorbance, flow rate of 1.0 ml/min).

Molar activities (decay corrected to the EOS) were determined with a calibration curve and the non-radioactive standard rhPSMA-7.3 · TFA, using an isocratic HPLC method (23% ACN/77% 100 mM ammonium format buffer, not pH adjusted, flow rate 1.0 ml/min, at the analytic HPLC system), using chromatograms obtained at 240 nm as an appropriate maximum of UV absorbance.

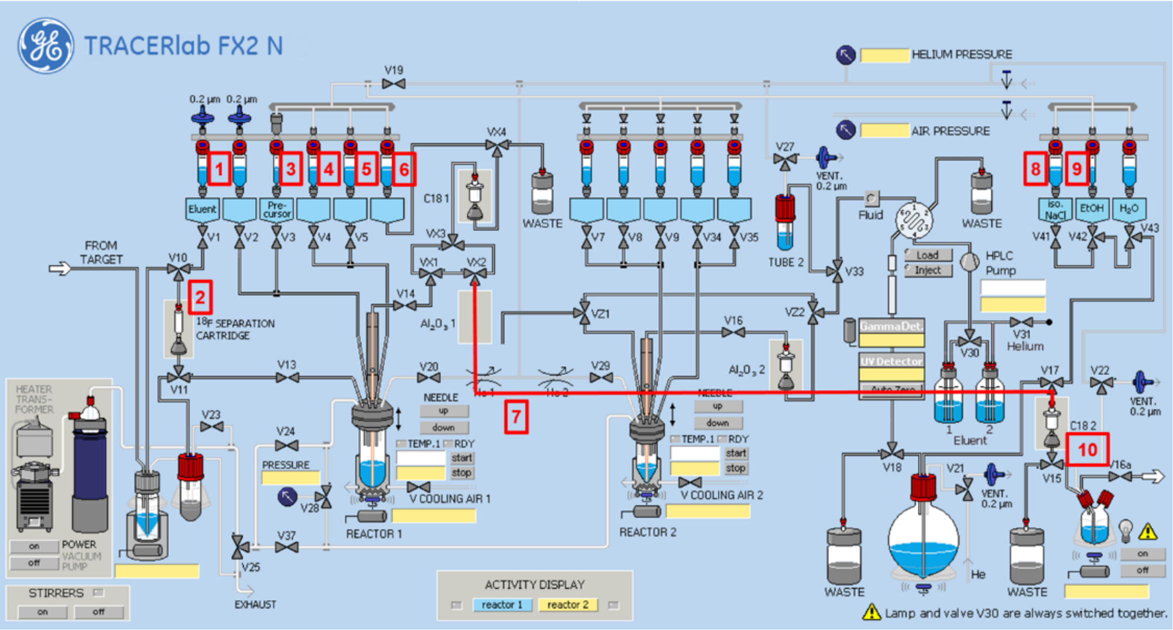

FIGURE S9: Synthesis module FX2 N (GE Healthcare) with the hardware modifications for the radiosynthesis of [^{18}F]rhPSMA-7.3 (entry 1: 2.0 ml K_2CO_3/K_{222} ACN/water solution; entry 2: QMA cartridge; entry 3: rhPSMA-7.3 in 1.0 ml DMSO and 8.5 µl conc. acetic acid; entry 4: 10 ml 0.1 M aq. NaOAc buffer (pH = 5); entry 5: 10 ml 0.1 M aq. NaOAc buffer (pH = 5); entry 6: 5 ml 0.1 M aq. NaOAc buffer (pH = 5); entry 7: inlet connection Al_2O_3 1 to the C18 cartridge at the position C18 2; entry 8: 1.0 ml 50% aq. EtOH; entry 9: 1.0 ml 50% aq. EtOH; entry 10: HLB cartridge).

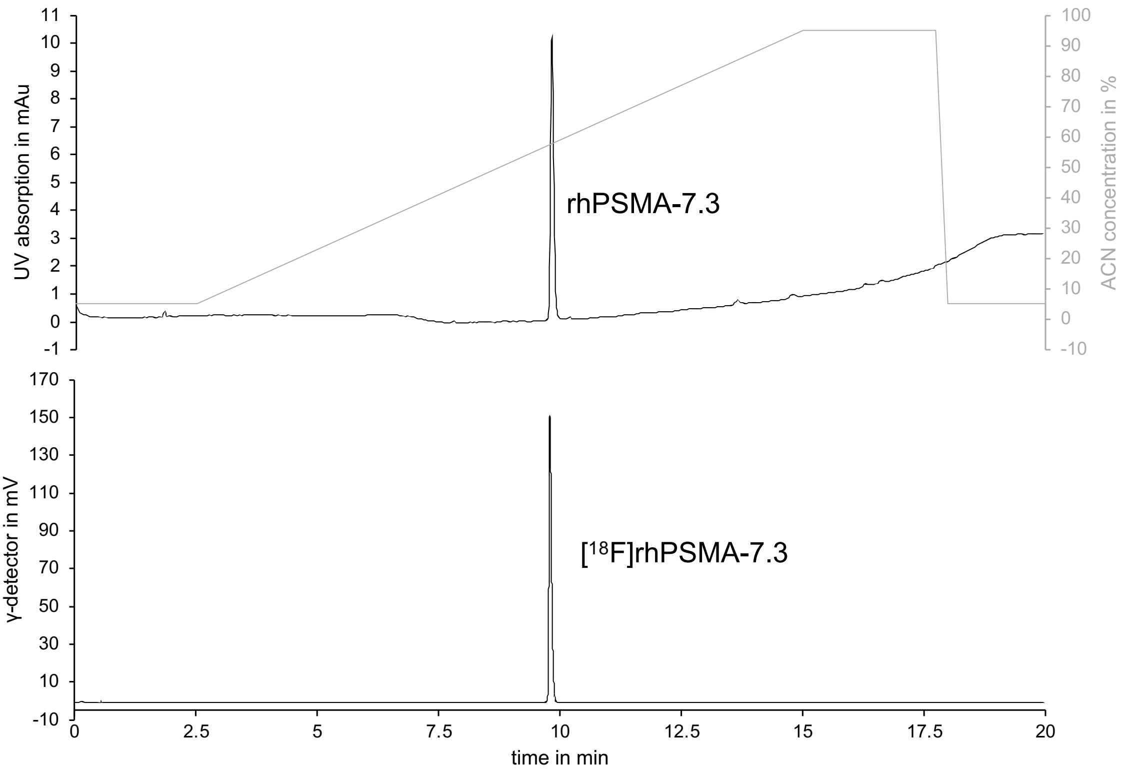

FIGURE S10: Analytical UV- and radio-HPLC chromatograms of the final product [^{18}F]rhPSMA-7.3 (conditions: linear-gradient of ACN/0.1 M aq. ammonium format (pH = 6; grey) with a flow rate of 1.0 ml/min; at 240 nm)

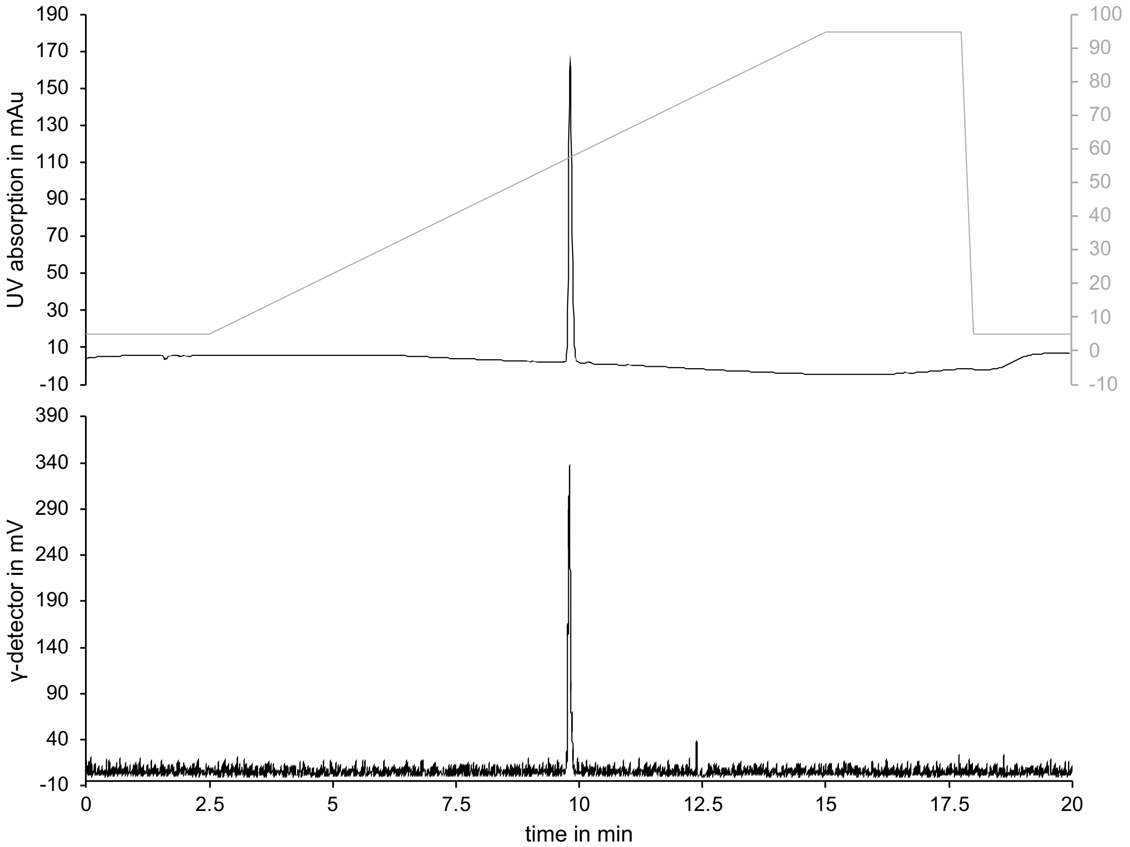

FIGURE S11: Analytical UV- and radio-HPLC chromatograms of the final product [^{18}F]rhPSMA-7.3 spiked with the non-radioactive reference rhPSMA · TFA (conditions: linear-gradient of ACN/0.1 M aq. ammonium formate (pH = 6; grey) with a flow rate of 1.0 ml/min; at 240 nm)

S2.2 Cell culture

GL261-luc2 cells were cultured in Dulbecco's Modified Eagle Medium (DMEM), high glucose, with 10% fetal bovine serum and challenged every eighth passage with Geneticin (G418) for selection. The cells were harvested following treatment with trypsin, counted and resuspended in pure DMEM without serum at a concentration of 25000 cells/µl.

S2.3 PET/MR and ROI definition

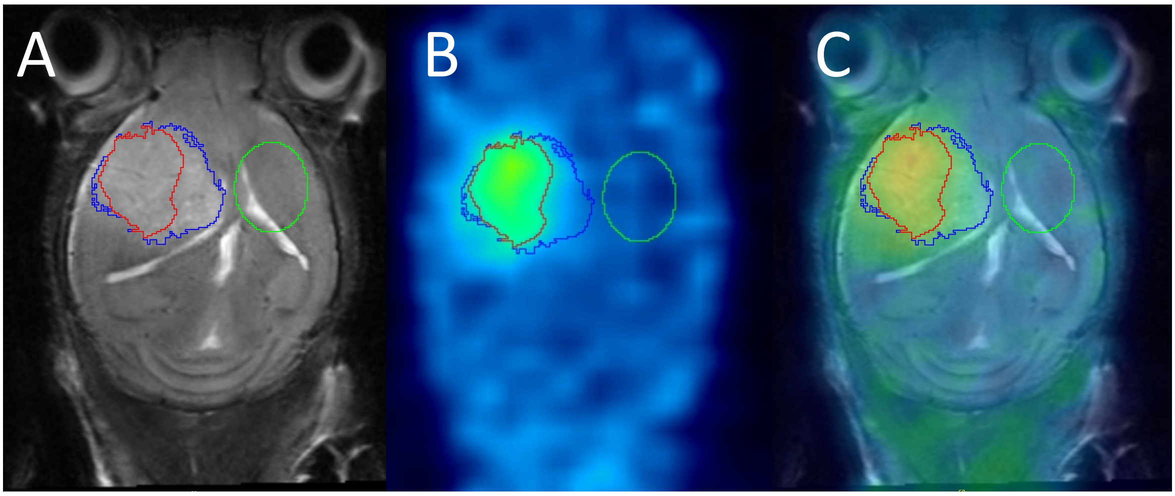

FIGURE S12: Tumor volume ROI placement. A: MRI, B: [^{18}F]fluciclovine PET and C: [^{18}F]fluciclovine PET/MRI. Blue: ROI based on MRI hyperintense signal, Red: 50% threshold ([^{18}F]fluciclovine) of blue ROI based on PET, Green: Control ROI in the contra lateral hemisphere.

Tumor MR monitoring was done with the following parameters:

- T2 weighted FSE (T_R: 3000 ms, T_E: 45 ms, 4 averages, slice thickness: 0.5 mm, matrix: 243, 18, 266, resolution: 0.0833, 0.5996, 0.0781)
- DWI (EPI, T_R: 5000 ms, T_E:27 ms, averages: 1, slice thickness: 0.7 mm, b = 0, 100, 200, 400, 600, 800, 1000, 4 directions, matrix: 171x128x119, resolution: 0.1172 isotropic)

Determination of number of animals was performed with G*Power using the following parameters:

Analysis: A priori: Compute required sample size

**Input:**	Tail(s)	=	Two
	Effect size d	=	2.5
	α err prob	=	0.1
	Power (1-β err prob)	=	0.9
	Allocation ratio N2/N1	=	1
**Output:**	Noncentrality parameter δ	=	3.5355339
	Critical t	=	1.9431803
	Df	=	6
	Sample size group 1	=	4
	Sample size group 2	=	4
	Total sample size	=	8
	Actual power	=	0.9276887

S2.4 *Ex vivo* Autoradiography

8.04 MBq [^{18}F]-rhPSMA-7.3 were i.v. injected in a 18.4 g C57Bl/6J mouse with GL261-luc2 brain tumor (33 days after inoculation). The mouse was sacrificed immediately after the last PET frame (60 min post i.v. [^{18}F]rhPSMA-7.3 injection). The brain was collected, snap frozen in liquid nitrogen and cryosectioned (16 µm slice thickness). The brain slices were fixed on super frost slides (Thermo Fisher Scientific Inc., Waltham, USA) and adhesive copper tape attached to the back of the slides for imaging with an online autoradiography system (BeaQuant, AI4R, Nantes, France). Autoradiograms were reconstructed to a resolution of 50 µm and analyzed by visual inspection using the BeaQuant v2.1.8 imaging software (AI4R, Nantes, France) as shown in figure 5A.

S2.7 *In vivo* stability of [^{18}F]FET and [^{18}F]rhPSMA-7.3

Plasma preparation

Healthy C57Bl/6J mice (19.3 ± 0.7 g, anesthetized with 1.8 % isoflurane) were i.v. (lateral tail vein) injected with 184.0 ± 61.0 MBq [^{18}F]FET (n=6) or 177.2 ± 33.6 MBq [^{18}F]rhPSMA-7.3 (n=6). Blood was collected by cardiac puncture at 30, 60, 90 min or 15, 30, 60 min p.i., [^{18}F]FET or [^{18}F]rhPSMA-7.3 resp. Whole blood was centrifuged (12000 rpm, 5 °C for 3 min) and plasma was separated.

Radiometabolite analysis of [^{18}F]rhPSMA-7.3

Plasma samples were diluted with water (plasma/water 1 : 9 v/v) and 10 µl of the dilution was administered to RP-C18 TLC plates F_{254} (ALUGRAM® RP-18W/UV254, Macherey-Nagel, Germany) with gentle heating (55 °C). The TLC plates were developed with a solvent mixture of ACN/0.5 M aq. sodium acetate buffer (pH = 5) (1 : 2 v/v). The developed TLC plates were dried and exposed for 0.1-5 min to a phosphor screen (Perkin Elmer Inc., USA) and imaged (Cyclone Plus, Perkin Elmer Inc., USA). Subsequently, the images were analyzed with ImageJ and visible peaks integrated to calculate the amount of intact radiotracer in comparison to radiometabolites.

Radiometabolite analysis of [^{18}F]FET

Protein precipitation of blood plasma samples was performed by adding an ice-cold mixture of acetone/water (9 : 1 v/v) in a ratio of plasma/extraction solvent of 1 : 4 (v/v). The samples were sonicated for 3 min at room temperature, equilibrated on ice for 3 min, sonicated for 3 min at room temperature, and 5 min centrifuged at 12000 rpm at 4 °C. After separation of the supernatant 1 from the protein pellet, 100 µl acetone/water (9 : 1 v/v) were added to the pellet. The sample was then 3 min sonicated (room temperature), 3 min equilibrated on ice, 3 min sonicated (room temperature), and 5 min centrifuged at 12000 rpm at 4 °C. Supernatant 2 was removed from the pellet and combined with supernatant 1. Aliquots (5 µl) were taken from raw plasma, supernatant 1 and 2, and were checked for their activity in a gamma counter (Wizard^2 1-Detector Gamma Counter, PerkinElmer Inc., USA). The combined supernatants were gently concentrated at 55 °C with a gentle flow of nitrogen to a final volume of ~100 µl. From this solution, ~10 µl were administered to silica gel G60 plates (with fluorescence indicator F_{254}, gentle heating of 55 °C and nitrogen flow) and developed with a solvent mixture of 8 : 2 : 0.1 ACN/0.1 M aq. ammonium formate buffer/conc. formic acid (v/v/v). The developed TLC plates were dried and exposed to a phosphor screen (0.1-5 min, Perkin Elmer Inc., USA) and imaged (Cyclone Plus, PerkinElmer Inc., USA). Subsequently, the images were analyzed with ImageJ and visible peaks integrated to calculate the amount of intact radiotracer in comparison to the radiometabolites.

For the calculation of the final extraction efficiencies (ExE), the supernatants samples from the first (A_{ex1}) and second extraction (A_{ex2}), and the pellet (A_p) were measured in a γ-counter. Activities of the samples were volume and decay corrected, and the following formula was applied to calculate the extraction efficiency (ExE):

$$ExE = \frac{A_{ex1} + A_{ex2}}{A_p + A_{ex1} + A_{ex2}} \cdot 100\%$$

S2.6 Western Blotting

Protein extraction from cell samples was performed at 4 °C in the presence of RIPA buffer supplemented with proteinases inhibitors (cOmplete ULTRA tablet, Roche, Prod.-Nr. 05892970001). Briefly, the suspension was incubated in a shaker for 20 min and, thereafter, centrifuged for 10 min at 14000 *g*. The extraction from tissue started with a mechanical grinding

in liquid nitrogen and posterior disruption in RIPA with proteinases inhibitors in the shaker for 30 min and sonication for 15 min at 4 °C. Followed with centrifugation for 10 min at 14000 *g*. The supernatants containing the proteins were collected and stored at –20 °C until the experiment. A sample size of 50 µg of protein was loaded per well into a SDS gel (Criterion TGX Stain-free precast gel, Bio-RAD, Prod.-Nr 5678033) and run into a Criterium electrophoresis system and Blotter (Bio-RAD). IBrigth™ pre-stained protein ladder and Magic MarK™ XP Western were used as molecular weight protein standards. The proteins were transferred to a nitrocellulose membrane after electrophoresis. 5% non-fat dried milk (NFDM) in TBS-T was used as blocking buffer. The rabbit monoclonal anti-PSMA (CST, Prod.-Nr 12815) was diluted 1 : 1000 in 5% BSA in TBS-T (following the manufacturer reconmendations) and incubated overnight at 4 °C in the rotator. Subsequently, the membrane was washed three times with TBS-T and incubated with HRP-goat anti-rabbit (abcam, Prod.-Nr ab205718) 1 : 5000 in the 5% NFDM at RT for 1h.SuperSignal WestPico Chemi-luminescent substrate kit (Thermo Scientific, Prod.-Nr 34578) was used for development.

S2.7 Fluorescence Immunohistochemistry

The formalin-fixed slides were deparaffinized in xylene, immersed in decreasing concentrations of ethanol, and rehydrated in water. Therefore, all sections were processed for heat-induced antigen retrieval immersed in 0.01 M sodium citrate buffer (pH 6.0) at 60 °C, overnight. Following several rinses in TBS, sections were blocked with TBS containing 3% bovine serum albumin (BSA) and 10% fetal bovine serum (FBS) for 2 h, at room temperature. The slides were washed twice with TBS/Triton X-100 (0.025 %) for 5 min, drained, and the liquid excess was wiped around the sections with tissue paper. The sections were incubated with rabbit monoclonal antibody anti-PSMA (1 : 100; Cell Signaling, cat. no. 12815) and anti-CD31 conjugated with phycoerythrin (1 : 100; Miltenyi, cat. no. 130-11-354) diluted in TBS/1% BSA overnight, at 4 °C. Therefore, slides were rinsed twice with TBS/Triton X (0.025%) for 5 min and incubated with anti-rabbit secondary antibody AlexaFluor 488 (1 : 500; ThermoFisher; cat. no. A32731) in TBS-BSA (1%) for 1 h, at room temperature. After several rinses in TBS, the sections were cover-slipped with a mounting medium with DAPI (Abcam, cat.no. ab104139). Microscopic images were captured by the ZeissAxio microscopy system (Zeiss, Oberkochen, Germany).

S3. Supplemental table and figures

S3.1 Radiochemistry

Table S1: Summary of radiosynthesis results of the applied radiotracers. (RCY - Radiochemical yield, d.c. EOS – decay corrected to the end of synthesis, A_m – molar activity, EOB – end of bombardment)

Feature	Radiotracers		
	[^{18}F]FET (n = 4)	[^{18}F]fluciclovine (n = 2)	[^{18}F]rhPSMA-7.3 (n = 5)
RCY (d.c. EOS)	52.0 ± 7.6%	37.8 ± 12.3%	36.6 ± 7.0%
RCP	> 99%	> 99%	> 99%
A_m or final activity per volume (all d. c. EOS)	123.8 ± 34.5 GBq/µmol; 0.8 ± 0.1 GBq/ml	n. a.; 0.6 ± 0.2 GBq/ml	10.5 ± 1.8 GBq/µmol; 2.9 ± 0.8 GBq/ml
Total synthesis time (EOB to EOS)	67 ± 1 min	85 ± 3 min	62 ± 3 min
Total final product activity (EOS)	4.3 ± 1.0 GBq	2.2 ± 0.7 GBq	3.1 ± 0.8 GBq
pH of the final formulation	7.5 ± 0.1	6.2 ± 0.9	7.2 ± 1.0 (n = 4)

S3.2 In vivo stability of [18F]FET and [18F]rhPSMA-7.3

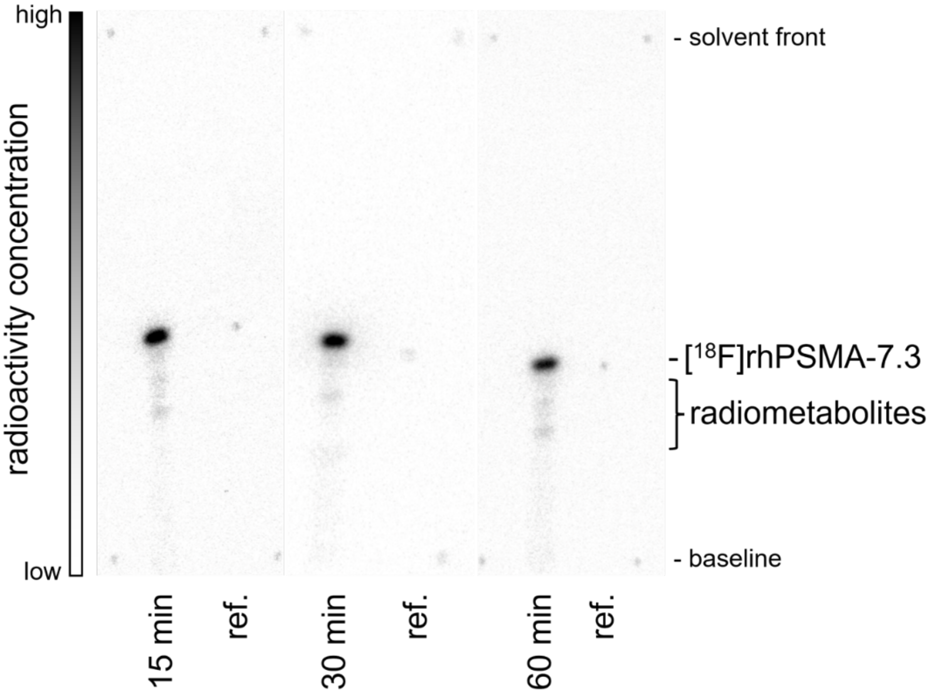

FIGURE S13: Examples of autoradiography images of the radio-TLC's from mouse plasma samples 15, 30, and 60 min p.i. of [18F]rhPSMA-7.3 (conditions: RP-C18 TLC plates F_{254}; mobile phase 1 : 2 ACN/0.5 M aq. sodium acetate buffer (pH = 5) (v/v)).

4

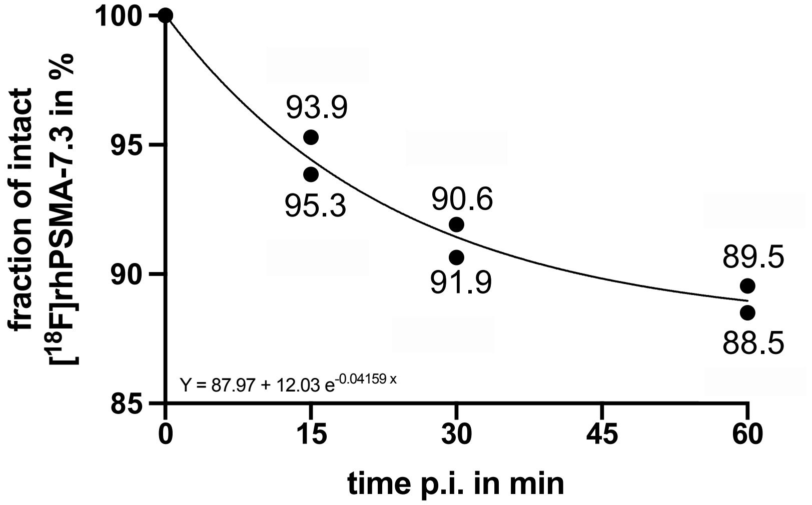

FIGURE S14: Dynamic data of intact [18F]rhPSMA-7.3 based on radio-TLC investigation of mouse plasma samples (n=2/time point). Single values and curve function used for metabolite correction in pharmacokinetic modeling.

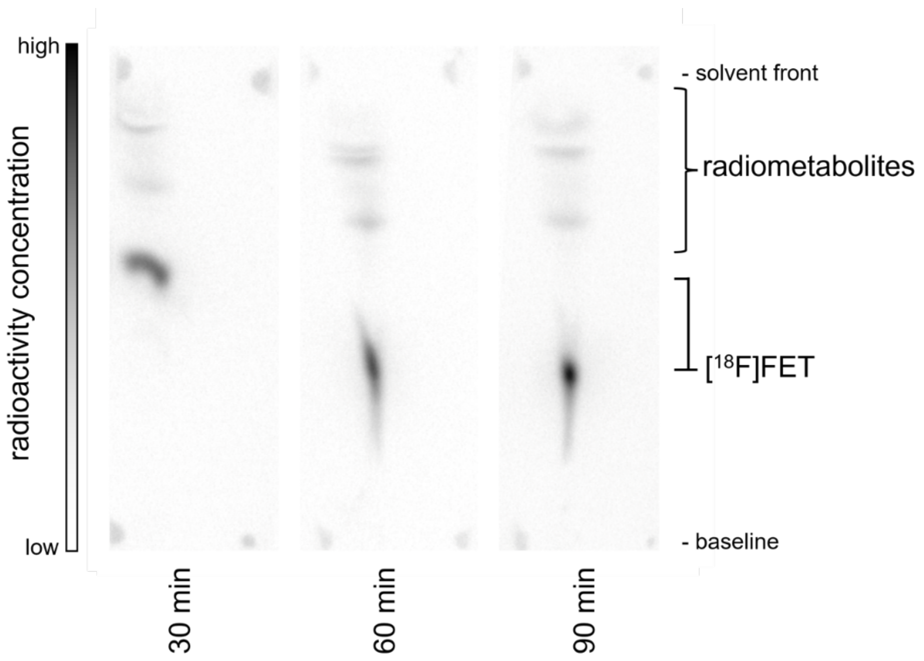

FIGURE S15: Examples of autoradiography images of the radio-TLC's from mouse plasma samples 30, 60, and 90 min p.i. of [^{18}F]FET (conditions: silica gel G60 plates F_{254}; mobile phase 8 : 2 : 0.1 ACN/0.1 M aq. ammonium formate buffer/conc. formic acid (v/v/v)).

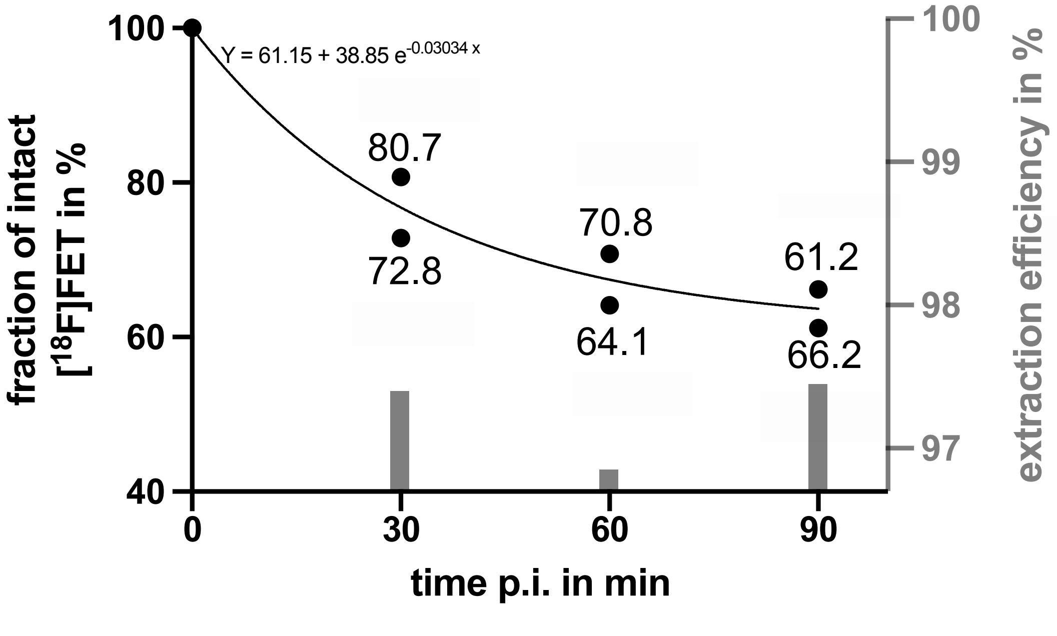

FIGURE S16: Dynamic data of intact [^{18}F]FET based on radio-TLC investigation of mouse plasma (n=2/time point) after protein precipitation. Single values and curve function used for metabolite correction in pharmacokinetic modeling. In grey: extraction efficiencies are shown as average of the two plasma extractions for each time point.

S3.3 Diffusion weighted imaging – ADC maps

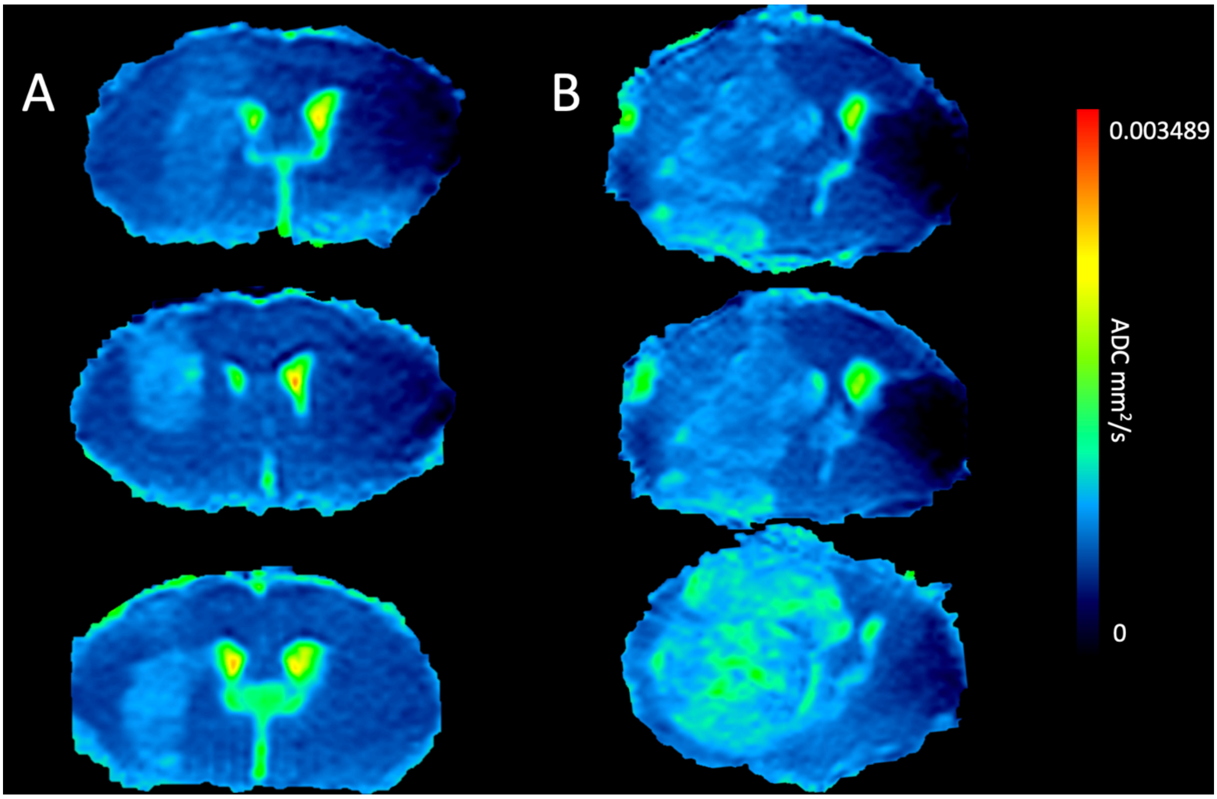

FIGURE S17: ADC maps of three representative animals at A) 21 days and B) 28 days post-surgery.

S3.4 Graphical analysis (28 days). Logan and Patlak plots to differentiate reversibility

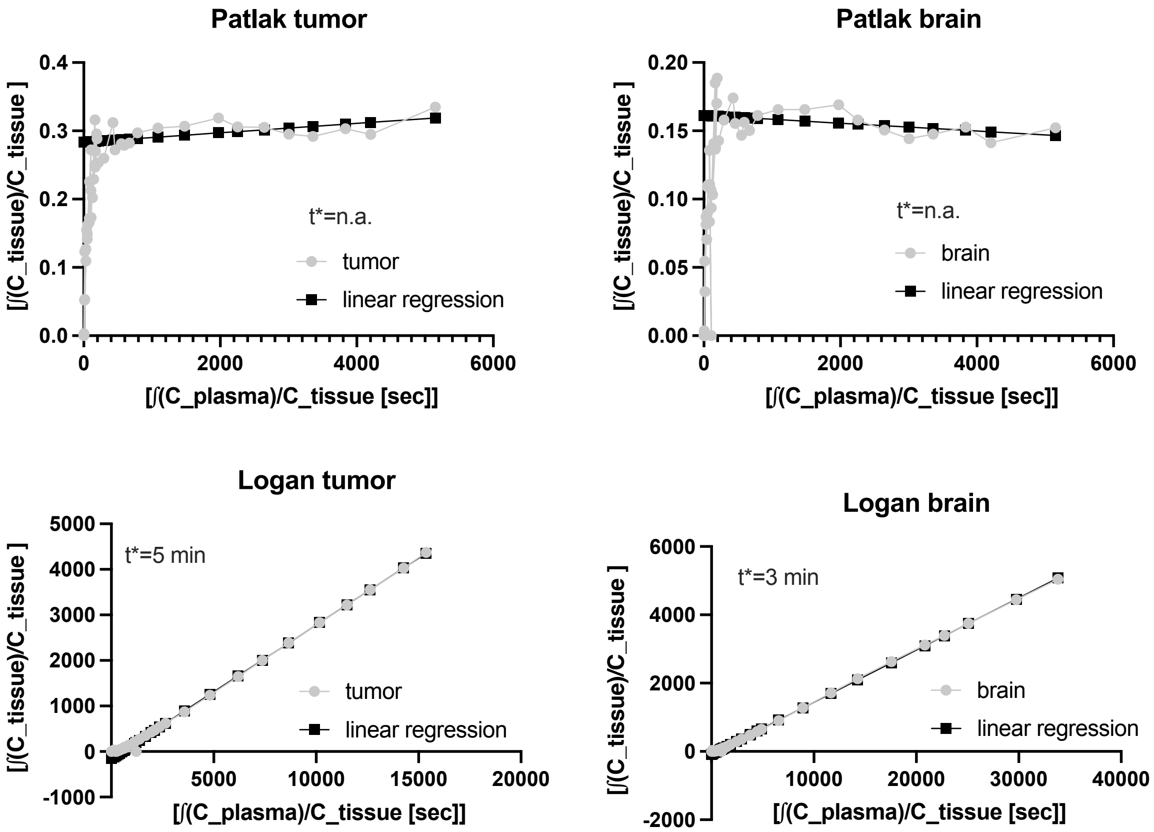

FIGURE S18: Patlak and Logan analysis in tumor and brain following i.v. injection of $[^{18}F]$rhPSMA-7.3. Slope obtained by fitting the last part of the respective plot (squares) while the slope represents Ki or Vt, resp. Data of one representative animal at 28 days post tumor cell inoculation. The Patlak plots show no linear fitting pattern among the animals with higher deviations for tumor ($R^2 = 0.32$) and the healthy brain region ($R^2 = 0.69$). Furthermore, the curve expresses a small (for brain negative) slope (Ki) close to zero (mean, tumor: $-6.25208*10^{-5}$ or brain: $-5.31*10^{-4}$ ml/ccm/min) and hence no relevant uptake of an irreversible compartment is present. Contrary, the reversible Logan model reached linearity very fast and within minutes following the injection for both brain and tumor ($R^2 = 0.9999$) indicating a reversible binding pattern with Vt significant ($p<<0.001$) higher for tumor (0.24) then for healthy brain (0.11). Table S2 and S3.

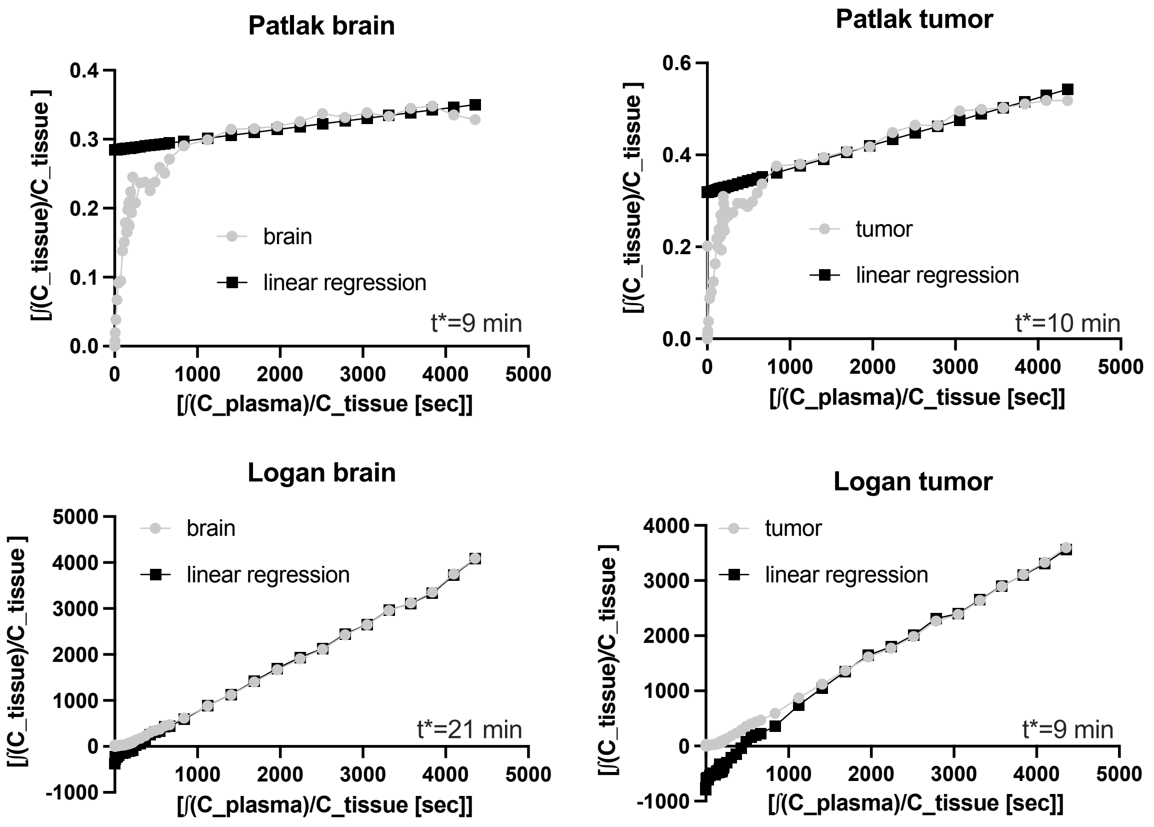

FIGURE S19: [18F]FET graphical analysis for one representative animal. The Patlak plots express deviation from linearity at earlier time points < 10 min (mean, tumor: 7 min, brain: 6 min) indicating, non/low irreversible binding or trapping. However, the deviations of the regression curve ($R^2 = 0.70$) indicate the existence of a dominating reversible part. Furthermore, Logan plots reach linearity at later time points but with more accuracy ($R^2 = 0.998$), which confirms reversible binding kinetics of [18F]FET in brain and tumor tissue.

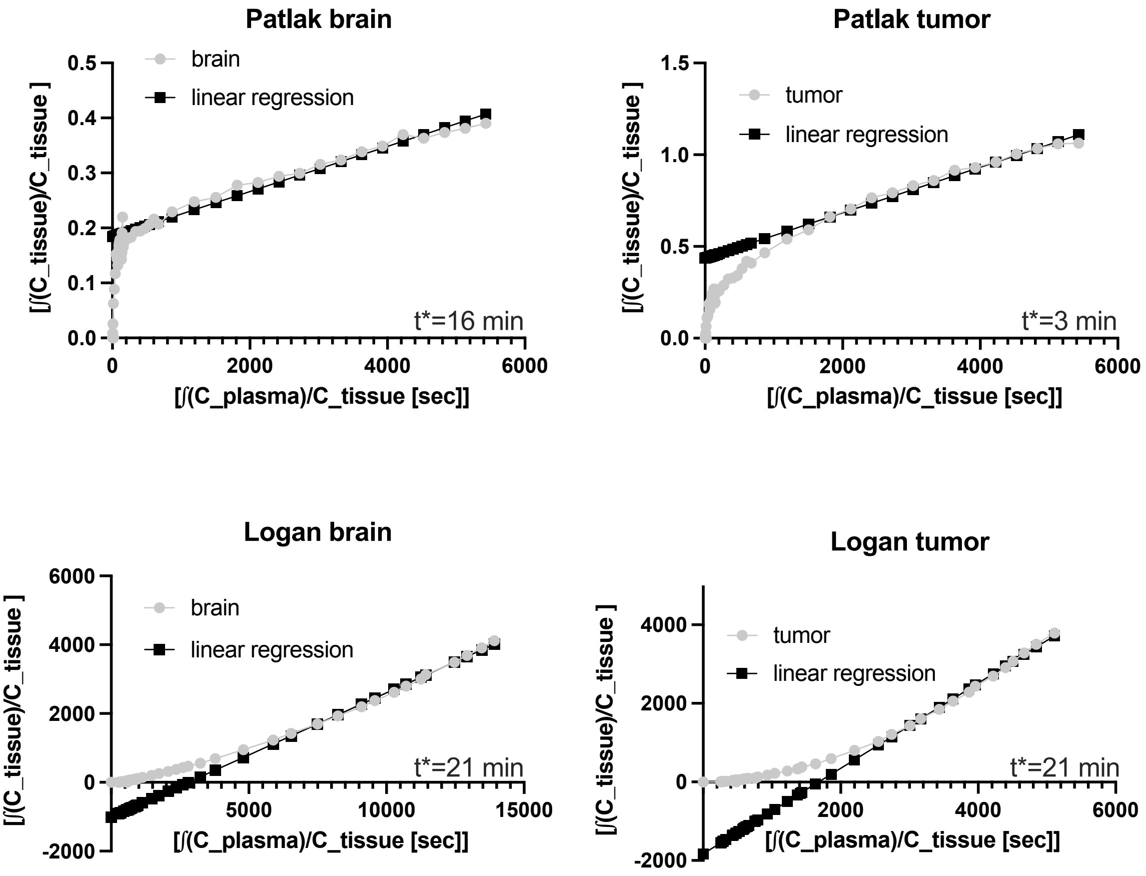

FIGURE S20: [^{18}F]fluciclovine graphical analysis for a representative animal. Applying the Patlak analysis, linearity starts with high inter animal variability at time points from 3 - 30 min (mean, tumor: 18 min, brain: 10 min). However, the linear part indicates irreversible uptake during the scanning time. Logan plots reach linearity more stable between 20 and 30 min (mean, tumor: 26 min, brain: 22 min) but also with more accuracy ($R^2 = 0.996$), confirming reversible binding kinetics of [^{18}F]fluciclovine. [^{18}F]FET and [^{18}F]fluciclovine, both radiotracers express similar kinetic uptake patterns with regard to Patlak and Logan plot analysis. Concordantly with the kinetic modeling results from the 2TCM, [^{18}F]fluciclovine shows higher mean Vt (1.23 vs. 0.78) in tumor as well as higher Ki (0.008 vs. 0.003), while the uptake kinetics of the healthy brain are almost identical (Vt: 0.4 vs 0.5) (Ki: 0.002 vs 0.001) confirming the observed increased TBR for [^{18}F]fluciclovine.

Table S2: Kinetic parameters (mean) 28 days after tumor cell inoculation

		K1 (ml/cm³/min)	k2 (min⁻¹)	k3 (min⁻¹)	k4 (min⁻¹)	Vs	Vt	AIC	SC	MSC
[18F]FET										
tumor	2TCM	0.19	0.26	0.03	0.04	0.70	1.38	78.11	85.64	3.06
contr lat		0.21	0.33	0.02	2.03	0.17	0.71	130.83	138.35	1.49
t test p value		0.47	0.14	0.17	0.18	0.05	0.04			
[18F]FET										
tumor	1TCM	0.17	0.14				1.01	101.94	105.71	2.57
contr lat		0.20	0.24	-	-	-	0.63	133.31	137.07	1.44
T.Test p value		0.42	0.14				0.13			
[18F]FET		Vt Logan		Ki Patlak						
tumor		0.88		4.8E-03						
contra lat		0.65		0.39						
T.Test p value		0.07		0.02						
[18F]fluciclovine										
tumor		0.16	0.63	0.09	0.02	1.26	1.50	57.23	64.80	3.94
contra lat	2TCM	0.17	1.03	0.04	0.02	0.33	0.48	94.33	101.89	1.81
T.Test p value		0.43	0.061	0.0007	0.33	0.0001	0.00004			
[18F]fluciclovine										
tumor	1TCM	0.05	0.05				1.05	141.49	145.28	2.22
contra lat		0.09	0.31	-	-	-	0.29	149.80	153.58	0.68

T.Test p value		0.064	0.001				0.0001			
[18F]fluciclovine		Vt Logan		Ki Patlak						
tumor		1.23		8.22E-03						
contra lat		0.40		2.37E-03						
T.Test p value										
[18F]rhPSMA-7.3										
tumor		0.28	1.18				0.24	96.9	100.12	1.53
contra lat	1TCM	0.08	0.84	-	-	-	0.09	117.67	120.91	1.25
T.Test p value		0.01	0.12				0.0006			
[18F]rhPSMA-7.3										
tumor		0.34	2.6	0.6	0.47	0.07	0.24	95.29	101.12	1.58
contra lat	2TCM	0.08	1.09	0.15	2.82	0.01	0.1	124.17	130.08	1.17
T.Test p value		0.0012	0.11	0.31	0.04	0.34	0.0017			
[18F]rhPSMA-7.3		Vt Logan		Ki Patlak						
tumor		0.26		1.9E-04						
contra lat		0.12		-4.1E-04						
T.Test p value		0.00027								

Table S3: Kinetic parameters (mean) 21 days after tumor cell inoculation

		K1 ($ml/cm^3/min$)	k2 (min^{-1})	k3 (min^{-1})	k4 (min^{-1})	Vs	Vt	AIC	SC	MSC
$[^{18}F]$FET										
tumor	2TCM	0.55	0.94	0.01	1.35	2.0E+202	2.0E+202	138.8	145.3	1.0
contr lat		0.15	0.54	0.05	0.09	0.12	0.37	139.1	145.6	0.93
T.Test p value	0.08	0.07	0.16	0.06	0.18	-	-			
$[^{18}F]$FET										
tumor	1TCM	0.29	0.52				0.51	137.7	141.33	1.01
contr lat		0.13	0.33	-	-	-	0.40	139.8	143.4	0.90
T.Test p value	0.07	0.13	0.21				0.04			
$[^{18}F]$FET		Vt Logan		Ki Patlak						
tumor		0.6		3.3E-03						
contra lat		0.45		1.8E-03						
T.Test p value		0.013		0.19						
$[^{18}F]$fluciclovine										
tumor		0.23	0.49	0.02	0.0045	-	-	121.4	128.97	1.73
contra lat	2TCM	0.07	0.41	0.03	0.0043	-	-	136.23	134.06	1.27
T.Test p value		0.14	0.38	0.24	0.49					
$[^{18}F]$fluciclovine										
tumor	1TCM	0.14	0.17				0.84	162.78	166.56	0.89

contra lat		0.04	0.1	-	-	-	0.4	166.92	170.71	0.64
T.Test p value		0.14	0.25				0.00005			
[^{18}F]fluciclovine		Vt Logan		Ki Patlak						
tumor		1.12		7.51E-03						
contra lat		0.63		3.67E-03						
T.Test p value		0.013								
[^{18}F]rhPSMA-7.3										
tumor		0.10	0.84				0.13	133.35	138.87	1.21
contra lat	1TCM	0.04	0.5	-	-	-	0.08	158.19	161.70	0.60
T.Test p value		0.004	0.071				0.0592			
[^{18}F]rhPSMA-7.3										
tumor		0.07	0.70	0.01	5.3	0.02	0.14	138.78	144.78	1.16
contra lat	2TCM	0.04	0.52	0.005	5.55	0.005	0.11	173.50	179.50	0.26
T.Test p value		0.18	0.22	0.31	0.50	0.25	0.29			
[^{18}F]rhPSMA-7.3		Vt Logan		Ki Patlak						
tumor		0.19		-6.34E-04						
contra lat		0.09		-4.60E-04						
T.Test p value		0.017								

TABLE S4: Kinetic parameters of the tumor region using the simplified reference tissue model [25] (SRTM) with the contra lateral region as reference. (BPnd – binding potential none-displaceable; BPnd TCM – binding potential non-displaceable derived from V_T of the 2-tissue-compartment model)

		28 d	21 d
[^{18}F]FET	BPnd	0.8	0.19
	BPnd TCM	0.54	0.67
[^{18}F]fluciclovine	BPnd	1.93	1.34
	BPnd TCM	0.32	0.48
[^{18}F]rhPSMA-7.3	BPnd	1.31	0.88
	BPnd TCM	0.36	0.62

To compare the results of the 2TCM and the SRTM the calculation as published by Koopman et al. [26] was applied. Briefly, the distribution volume ratio (DVR) was calculated by dividing the V_T of the tumor region by the V_T of the reference region (resp. region at the contra lateral hemisphere). To receive the BPnd the reciprocal of the DVR was used which equals the BPnd TCM in TABLE S4.

S3.5 PET kinetic modeling – parameter mapping

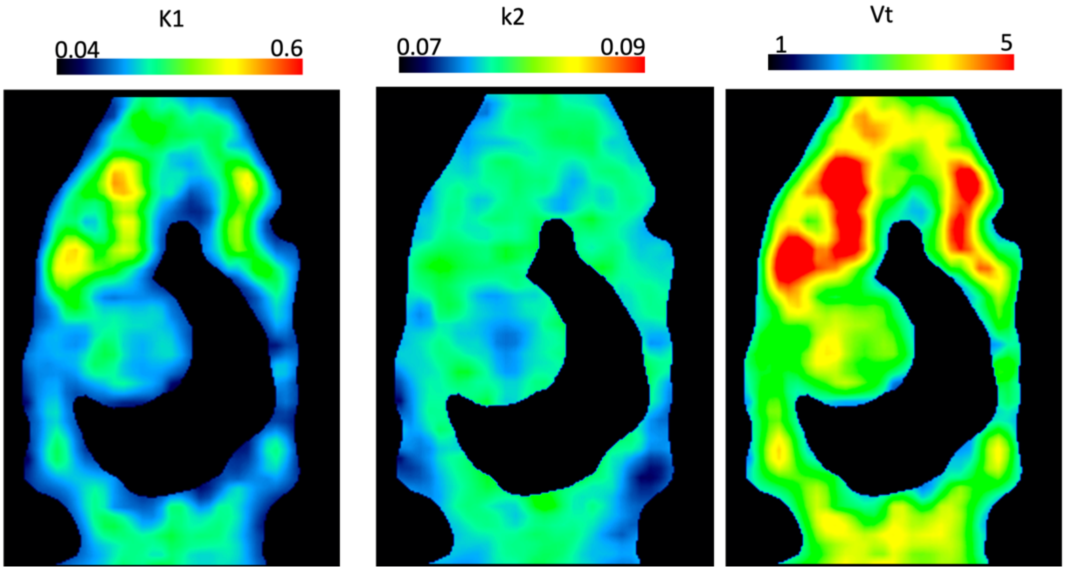

FIGURE S21: Parametric maps (2TCM) of a representative animal 28 days post - tumor cell inoculation after injection of [^{18}F]rhPSMA-7.3. Of note, due to low signal k3 and k4 maps are not presented here.

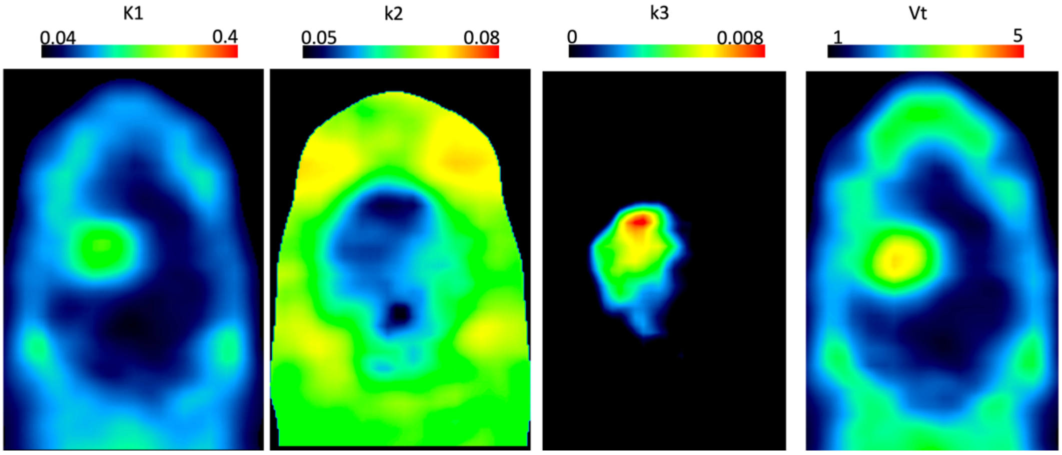

FIGURE S22: Parametric maps (2TCM) of a representative animal 28 days post - tumor cell inoculation after injection of [^{18}F]fluciclovine. Of note, due to no signal k4 maps are not presented here.

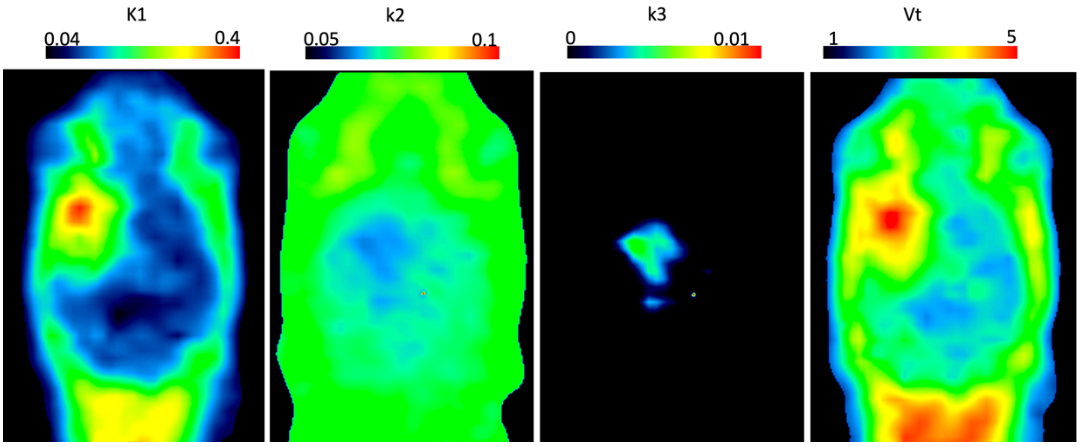

FIGURE S23: Parametric maps (2TCM) of a representative animal 28 days post - tumor cell inoculation after injection of [^{18}F]FET. Of note, due to no signal k4 maps are not presented here.

S4. Supplemental references

1. Bennett SR, Cruickshank G, Lindenmeyer A, Morris SR. Investigating the impact of headaches on the quality of life of patients with glioblastoma multiforme: a qualitative study. BMJ Open. 2016;6:e011616.

2. Oberheim Bush NA, Hervey-Jumper SL, Berger MS. Management of Glioblastoma, Present and Future. World Neurosurg. 2019;131:328–38.

3. Brandes AA, Tosoni A, Franceschi E, Reni M, Gatta G, Vecht C. Glioblastoma in adults. Crit Rev Oncol Hematol. 2008;67:139–52.

4. DeVita VT, Lawrence TS, Rosenberg SA. Cancer: principles & practice of oncology: primer of the molecular biology of cancer. Lippincott Williams & Wilkins; 2012.

5. Szopa W, Burley TA, Kramer-Marek G, Kaspera W. Diagnostic and Therapeutic Biomarkers in Glioblastoma: Current Status and Future Perspectives. Buonaguro FM, editor. Biomed Res Int. Hindawi; 2017;2017:8013575.

6. Fujita Y, Sasayama T, Tanaka K, Kyotani K, Nagashima H, Kohta M, et al. DWI for Monitoring the Acute Response of Malignant Gliomas to Photodynamic Therapy. Am J Neuroradiol. 2019;40:2045 LP – 2051.

7. Kinoshita M, Hashimoto N, Goto T, Kagawa N, Kishima H, Izumoto S, et al. Fractional anisotropy and tumor cell density of the tumor core show positive correlation in diffusion tensor magnetic resonance imaging of malignant brain tumors. Neuroimage. Elsevier; 2008;43:29–35.

8. Jain R. Measurements of tumor vascular leakiness using DCE in brain tumors: clinical applications. NMR Biomed. Wiley Online Library; 2013;26:1042–9.

9. Larsson HBW, Courivaud F, Rostrup E, Hansen AE. Measurement of brain perfusion, blood volume, and blood-brain barrier permeability, using dynamic contrast-enhanced T1-weighted MRI at 3 tesla. Magn Reson Med An Off J Int Soc Magn Reson Med. Wiley Online Library; 2009;62:1270–81.

10. Kostakoglu L, Agress H, Goldsmith SJ. Clinical Role of FDG PET in Evaluation of Cancer Patients. RadioGraphics. Radiological Society of North America; 2003;23:315–40.

11. Niyazi M, Geisler J, Siefert A, Schwarz SB, Ganswindt U, Garny S, et al. FET–PET for

malignant glioma treatment planning. Radiother Oncol. 2011;99:44–8.

12. Isselbacher KJ. Sugar and amino acid transport by cells in culture—differences between normal and malignant cells. N Engl J Med. Mass Medical Soc; 1972;286:929–33.

13. Vander Borght T, Asenbaum S, Bartenstein P, Halldin C, Kapucu Ö, Van Laere K, et al. EANM procedure guidelines for brain tumour imaging using labelled amino acid analogues. Eur J Nucl Med Mol Imaging. 2006;33:1374–80.

14. Albert NL, Weller M, Suchorska B, Galldiks N, Soffietti R, Kim MM, et al. Response Assessment in Neuro-Oncology working group and European Association for Neuro-Oncology recommendations for the clinical use of PET imaging in gliomas. Neuro Oncol. 2016;18:1199–208.

15. Michaud L, Beattie BJ, Akhurst T, Dunphy M, Zanzonico P, Finn R, et al. ^{18}F-Fluciclovine (^{18}F-FACBC) PET imaging of recurrent brain tumors. Eur J Nucl Med Mol Imaging. 2020;47:1353–67.

16. Langen KJ, Hamacher K, Weckesser M, Floeth F, Stoffels G, Bauer D, et al. O-(2-[^{18}F]fluoroethyl)-l-tyrosine: uptake mechanisms and clinical applications. Nucl. Med. Biol. Elsevier; 2006. p. 287–94.

17. Bolcaen J, Lybaert K, Moerman L, Descamps B, Deblaere K, Boterberg T, et al. Kinetic Modeling and Graphical Analysis of ^{18}F-Fluoromethylcholine (FCho), ^{18}F-Fluoroethyltyrosine (FET) and ^{18}F-Fluorodeoxyglucose (FDG) PET for the Fiscrimination between High-Grade Glioma and Radiation Necrosis in Rats. PLoS One. Public Library of Science; 2016;11:e0161845.

18. Jansen NL, Graute V, Armbruster L, Suchorska B, Lutz J, Eigenbrod S, et al. MRI-suspected low-grade glioma: is there a need to perform dynamic FET PET? Eur J Nucl Med Mol Imaging. Springer; 2012;39:1021–9.

19. Hutterer M, Nowosielski M, Putzer D, Jansen NL, Seiz M, Schocke M, et al. [18F]-fluoro-ethyl-l-tyrosine PET: a valuable diagnostic tool in neuro-oncology, but not all that glitters is glioma. Neuro Oncol. 2013;15:341–51.

20. Jansen NL, Suchorska B, Wenter V, Schmid-Tannwald C, Todica A, Eigenbrod S, et al. Prognostic Significance of Dynamic ¹⁸F-FET PET in Newly Diagnosed Astrocytic High-Grade Glioma. J Nucl Med. 2015;56:9 LP – 15.

21. Bourdier T, Greguric I, Roselt P, Jackson T, Faragalla J, Katsifis A. Fully automated one-pot radiosynthesis of O-(2-[18F]fluoroethyl)-l-tyrosine on the TracerLab FXFN module. Nucl Med Biol. 2011;38:645–51.

22. Svadberg A, Ryan O, Smeets R. PREPARATION OF F-18-FLUCICLOVINE, WO2014023775A1 [Internet]. WO2014023775A1; 2014. Available from: WO2014023775A1

23. Dyrstad, Knut R, Wickstrom T, Rajanayagam T. NOVEL FORMULATION AND METHOD OF SYNTHESIS, WO2016001199A1. 2016.

24. Wurzer A, DiCarlo D, Schmidt A, Beck R, Eiber M, Schwaiger M, et al. Radiohybrid ligands: a novel tracer concept exemplified by ¹⁸F- or ⁶⁸Ga-labeled rhPSMA-inhibitors. J Nucl Med [Internet]. 2019;jnumed.119.234922. Available from: http://jnm.snmjournals.org/content/early/2019/12/20/jnumed.119.234922.abstract

25. Wu Y, Carson RE. Noise reduction in the simplified reference tissue model for neuroreceptor functional imaging. J Cereb Blood Flow Metab. SAGE Publications Sage UK: London, England; 2002;22:1440–52.

26. Koopman T, Verburg N, Schuit RC, Pouwels PJW, Wesseling P, Windhorst AD, et al. Quantification of O-(2-[^{18}F]fluoroethyl)-L-tyrosine kinetics in glioma. EJNMMI Res. 2018;8:72.
